# Pheromone Recognition and Selectivity by ComR Proteins among *Streptococcus* Species

**DOI:** 10.1371/journal.ppat.1005979

**Published:** 2016-12-01

**Authors:** Erin Shanker, Donald A. Morrison, Antoine Talagas, Sylvie Nessler, Michael J. Federle, Gerd Prehna

**Affiliations:** 1 Department of Medicinal Chemistry and Pharmacognosy, University of Illinois at Chicago, Chicago, IL, United States of America; 2 Center for Biomolecular Science, University of Illinois at Chicago, Chicago, IL, United States of America; 3 Department of Biological Sciences, University of Illinois at Chicago, Chicago, IL, United States of America; 4 Institute of Integrative Biology of the Cell (I2BC), CEA, CNRS, Univ Paris-Sud, Université Paris-Saclay, France; 5 Center for Structural Biology, Research Resources Center, University of Illinois at Chicago, Chicago, IL, United States of America; 6 Department of Microbiology and Immunology, University of Illinois at Chicago, Chicago, IL, United States of America; National Jewish Health, UNITED STATES

## Abstract

Natural transformation, or competence, is an ability inherent to bacteria for the uptake of extracellular DNA. This process is central to bacterial evolution and allows for the rapid acquirement of new traits, such as antibiotic resistance in pathogenic microorganisms. For the Gram-positive bacteria genus *Streptococcus*, genes required for competence are under the regulation of quorum sensing (QS) mediated by peptide pheromones. One such system, ComRS, consists of a peptide (ComS) that is processed (XIP), secreted, and later imported into the cytoplasm, where it binds and activates the transcription factor ComR. ComR then engages in a positive feedback loop for the expression of ComS and the alternative sigma-factor SigX. Although ComRS are present in the majority of *Streptococcus* species, the sequence of both ComS/XIP and ComR diverge significantly, suggesting a mechanism for species-specific communication. To study possible cross-talk between streptococcal species in the regulation of competence, and to explore in detail the molecular interaction between ComR and XIP we undertook an interdisciplinary approach. We developed a ‘test-bed’ assay to measure the activity of different ComR proteins in response to cognate and heterologous XIP peptides *in vivo*, revealing distinct ComR classes of strict, intermediate, and promiscuous specificity among species. We then solved an X-ray crystal structure of ComR from *S*. *suis* to further understand the interaction with XIP and to search for structural features in ComR proteins that may explain XIP recognition. Using the structure as a guide, we probed the apo conformation of the XIP-binding pocket by site-directed mutagenesis, both in test-bed cultures and biochemically *in vitro*. In alignments with ComR proteins from other species, we find that the pocket is lined by a variable and a conserved face, where residues of the conserved face contribute to ligand binding and the variable face discriminate among XIP peptides. Together, our results not only provide a model for XIP recognition and specificity, but also allow for the prediction of novel XIP peptides that induce ComR activity.

## Author Summary

Bacteria transmit chemical signals to each other in a process known as quorum sensing. This adaptation is central to pathogenesis as it allows bacteria to coordinate a group response, such as when to form a biofilm, secrete virulence factors, or uptake new DNA. Quorum sensing in many species of Gram-positive bacteria utilizes secreted short peptide signals that are sensed by protein receptors that alter gene expression. The ComR-ComS receptor-signal pair is a regulator of competence for genetic transformation that is broadly conserved in a majority of *Streptococcus* species, including those pathogenic in humans and animals. Despite this conservation, we observe sequence diversity among ComRS orthologs that raises questions relating to sensory specificity and the molecular mechanism of peptide recognition. To address this, we directly tested the possibility for signaling cross-talk and identified three general categories of ComR receptors displaying strict, intermediate, and promiscuous responses to heterologous peptides. To elucidate the molecular mechanism of receptor specificity we determined an X-ray crystal structure of ComR from *S*. *suis*. We observe a conserved face of the ligand pocket important for peptide binding and a variable face that functions in peptide specificity. Finally, basic criteria necessary for peptide responses were used to redesign active peptides from inactive templates.

## Introduction

Competence, or the ability of bacteria to import extracellular DNA, is a trait widely conserved across both Gram-negative and Gram-positive bacteria. This natural transformation process allows bacteria to acquire new genes that increase genetic diversity and fitness, such as the gain of antibiotic-resistance determinants [1]. Common among bacteria that are able to undergo natural transformation is a minimal set of genes enabling transport and integration of DNA by homologous recombination. Broad conservation of these genes, even among bacteria for which natural transformation has not been demonstrated in laboratory settings [2] provides a mechanistic explanation for the widespread evidence of horizontal gene transfer in bacteria, and strongly suggests their evolutionary importance.

Within the genus *Streptococcus*, incorporating both pathogenic and commensal species to humans and animals, and those useful in industrial applications [3], the core competence genes are tightly regulated and their expression is coordinated by quorum sensing (QS). Generally, QS is utilized by both Gram-positive and Gram-negative bacteria to organize a community-wide response to environmental stimuli. For example, pathogens use QS to time the expression and secretion of virulence factors during pathogenesis [4, 5]. For most QS signaling, a small molecule such as an N-acyl homoserine lactone [6] or a peptide [7] is used as the secreted chemical messenger, although inter-bacterial communication can also be accomplished though the interaction of secreted proteins [8]. Competence in *Streptococcus spp*, is regulated either by the ComCDE system [9] or by the subject of our study, the ComRS pathway [10].

The ComCDE pathway is utilized by members of the Mitis and Anginosus *Streptococcus* groups and its peptide pheromone is sensed by the bacteria through a two-component signal transduction pathway [9, 11]. In contrast, all other groups of *Streptococcus* (Pyogenic, Mutans, Bovis, and Salivarius) employ the ComRS system, in which the pheromone is imported into the cytoplasm to bind the transcriptional regulator directly. Although the details of these QS pathways differ, both induce the expression of *sigX*, the *Streptococcus* alternative sigma-factor and its regulon [7]. It is SigX that subsequently initiates transcription of the competence genes required for the incorporation of the newly-acquired DNA [12].

The gene products of *comRS* comprise the pheromone precursor and the receptor of the pheromone. In *Streptococcus mutans*, ComS is a 17 amino acid pre-peptide that undergoes proteolytic maturation and secretion from the cell by an uncharacterized pathway to produce the active 7 aa pheromone XIP (*s* *igX*-inducing peptide) [13, 14]. To be sensed by bacteria, XIP must reach the cytoplasm, and does so by way of generalized peptide transporters of the oligopeptide permease (Opp) family [10, 15]. Once in the cytoplasm, XIP directly binds to and activates ComR [10].

ComR proteins are members of the RNPP (Rap/NprR/PrgX/PlcR) family of QS regulators, including Rgg, which are prevalent throughout the *Lactobacillales* order [16, 17]. Members of this family share several biochemical and structural similarities, most importantly the ability to bind peptide pheromones to govern protein activity. However, the exact mechanisms by which they respond to bound pheromone can differ significantly. For example, NprR undergoes oligomerization from a dimer to a tetramer in response to peptide binding [18], whereas Rgg proteins are thought to remain as dimers in both apo- and ligand-bound forms [19]. ComR is predicted to contain an N-terminal helix-turn-helix (HTH) DNA binding domain (DBD) and a C-terminal tetratricopeptide repeat (TPR) domain, which contains the site of pheromone binding [19, 20]. Once activated, the ComR/XIP complex binds at the promoter regions of *comS* and *sigX*, thus producing a pheromone-dependent positive-feedback regulatory loop and activating the SigX regulon [10, 21].

**Fig 1:**
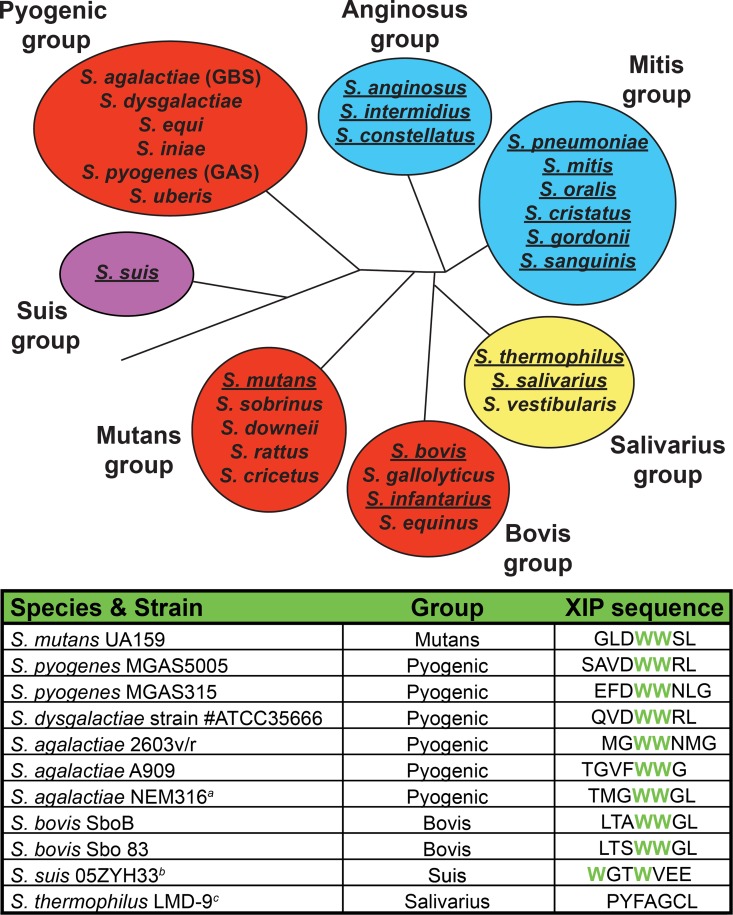
Distribution and conservation of competence pathways in *Streptococcus spp*. (Top) The Type-I ComRS pathway is found in the Salivarius group (yellow shading) and the ComCDE pathway in the Anginosus and Mitis groups (blue shading). The Type-II ComRS pathway is present in Bovis, Mutans, and Pyogenic groups of *Streptococcus* (red shading). The Suis group, or Type-III ComRS pathway (violet), contains attributes distinguishable from Type-I and Type-II. Underlined species indicate the demonstration of natural competence under laboratory conditions. (Bottom) The putative XIP sequences from the Type-II ComRS pathway demonstrate a conserved double tryptophan motif. Represented sequences were derived from small ORFs directly downstream of ComR and include the C-terminal 7 or 8 amino acids predicted to encode the mature pheromone. ^*a*^The ComR derived from *S*. *agalactiae* NEM316 was not investigated, however this species does encode a putative Type-II XIP. ^*b*^The *S*. *suis* 05ZYH33 XIP represents a Type-III XIP encoding two tryptophan residues (in green) interrupted by glycine and threonine. ^*c*^The *S*. *thermophilus* LMD-9 Type-I XIP, lacking a WW-motif, is included for reference.

There are three general classes of ComRS pathways, type-I (Salivarius), type-II (Bovis, Mutans, Pyogenic), and type-III (Suis) [10, 22]-[23]. The classification is based upon observed differences in both the *comS*/*sigX* promoters and XIP sequences, which in turn correspond with phylogenetic groupings (Fig 1). For example, the type-I and type-II *comS/sigX* promoter sequences differ within inverted repeats, at which ComR/XIP binds [10]. Additionally, the type-II *comS* genes encode XIP peptides containing a C-terminal WW-motif that is required for ComS and SigX activation in *S*. *mutans* UA159 [10], whereas this WW-motif is not found in type-I XIPs [22]. The type-III ComS contains two tryptophans similar to the type-II peptides, but differs in that the WW-motif is split by two residues [23]. As type-II ComRs represent the larger sample group and include pathogenic species, we chose to focus our study in these systems. Despite this classification and the broad conservation of type-II ComRS genes, there are significant sequence variations among both the XIP and ComR sequences. This observation and recent work suggesting communication between *Streptococcus* species [24] led us to investigate roles of these variations in intercellular communication, in particular XIP recognition.

To address the effect of these sequence varieties, we developed a ‘test-bed’ assay using *S*. *mutans* to measure the response of different type-II ComR proteins to various XIPs in bacterial culture. Not only did this reveal distinct degrees of specificity for XIP, but it also allowed demonstration of active ComRS systems that were only previously predicted. We also expanded this analysis to include the type-III representative, *S*. *suis*, to broaden our exploration of signaling peptide recognition. These experiments provided an initial survey of streptococcal species that are amenable to inter-species induction of competence. Additionally, we obtained an X-ray crystal structure of the ComR from *S*. *suis* and used this as a guide to probe the structural features of the apo conformation of the XIP binding-site by site-directed mutagenesis, both *in vivo* by the test bed assay and directly *in vitro* biochemically. The combined biochemical and structural data provide a model for how ComR discriminates between cognate and foreign pheromones for activation. Finally, we use our combined functional and structural data to predict and create new active peptide-pheromones.

## Results

### A ComR ‘test-bed’ system allows efficient and straightforward measurement of XIP-responsive transcription

**Fig 2:**
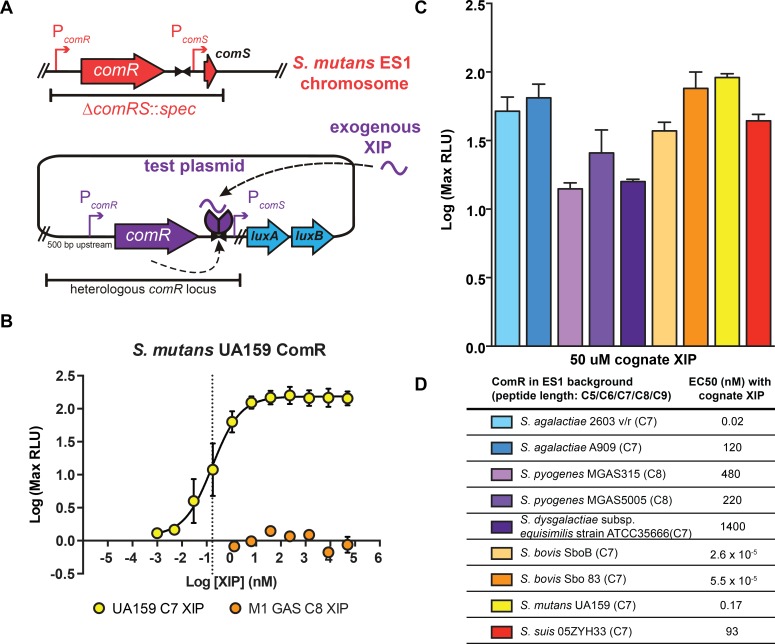
The *S*. *mutans* ‘test-bed’ to characterize ComR and cognate XIP activity of Bovis, Mutans, and Pyogenic species of *Streptococcus*. (A) The test-bed strain ES1 (*comRS*::*spec*) was used to host a plasmid containing an intact *comR* locus, including the *comR* gene (with 500 bp upstream), the conserved ComR DNA-binding site, and the *comS* promoter, from the selected *Streptococcus* strain. The *comS* promoter is positioned upstream from the *luxAB* luciferase reporter. (B) Luciferase activities of ES1 harboring the test plasmid containing the *S*. *mutans* UA159 ComR locus treated with synthetic UA159 (cognate) XIP-C7 or S. *pyogenes* MGAS5005 (non-cognate) XIP-C8. The dashed line indicates the EC_50_ value (88 nM) for UA159 XIP-C7. (C) Luciferase induction activity from cognate ComR–XIP treatments of each test strain, determined as maximum light activity responses to 50 μM cognate XIP compared to untreated cultures. (D) Cognate XIP EC_50_ values calculated from dose-responsive curves of each ComR (S1 Fig).

The entire known SigX regulon, as well as the proximal transcriptional regulator of *sigX*, ComR, is conserved throughout the Mutans, Bovis, and Pyogenic groups (Fig 1, top) [10]. Additionally, within these groups, the mature form of the peptide pheromone ComS contains a conserved double-tryptophan motif (‘WW’) (Fig 1, bottom) [10]. This led us to ask whether the putative ComRs encoded by members of these groups could respond to cognate and heterologous XIPs to activate *comS* and *sigX* transcription. To test this possibility, we developed a strain that would allow for control of the ComR and XIP variants present within the system. Previous findings showed that an *S*. *mutans* UA159 *ΔcomS* strain robustly activated luciferase genes expressed from the *sigX* promoter (P_*sigX*_::*luxAB*) upon addition of synthetic cognate XIP. Additionally, when a multi-copy plasmid carrying wild-type *comR* was provided to a *ΔcomR* strain, the ability to naturally transform exogenous genomic DNA was rescued [10]. With these observations in mind, we created an *S*. *mutan*s UA159 ‘test bed’ strain, ES1, in which the endogenous *comRS* locus was replaced with a spectinomycin-resistance cassette (Fig 2A). Because this strain contains neither *comS* nor *comR*, it is unable to produce or respond to XIP pheromones. A plasmid was used as a vehicle to provide different *comR* variants, under control of the native promoter, with adjoining *comR-comS* intergenic regions containing ComR binding sites and the *comS* promoter. The plasmid also contains the bacterial luciferase genes, *luxAB*, under transcriptional control of the cloned *comS* promoter. Clones harboring each *comR*-variant plasmid were constructed, grown, and treated with titrations of synthetic XIP peptides. Since productive interaction between ComR and XIP results in transcriptional activation of the *comS* promoter, we could test the functionality of ComR–XIP interactions by assessing the specific bioluminescent activity of each culture. To ensure that the test bed recapitulated observed results seen in wild-type *S*. *mutans* UA159, we assessed the UA159 ComR with serial dilutions of the cognate XIP-C7 (the C-terminal seven amino acids of ComS) and measured bioluminescence levels (Fig 2B). The observed EC_50_ value (effective concentration of XIP eliciting 50% of the log maximum luminescence) was 0.2 nM, with a maximal induction of 140-fold at 2 nM XIP (Fig 2B). These results confirmed that episomal expression of *comR* and exogenous provision of XIP in the test bed recapitulated reported observations in wild-type *S*. *mutans* [10, 25, 26].

### ComR proteins interact with their cognate XIPs to activate transcription from the *comS* promoter

To determine if other conserved ComRS pathways selected from the Bovis and Pyogenic groups also demonstrated ComR and XIP-dependent activity similar to *S*. *mutans* UA159, we developed a series of plasmids, each containing the analogous segment of DNA. These reporter plasmids were transferred into the ES1 background, allowing us to test whether the encoded ComR could activate the cognate P_*comS*_ in response to added synthetic cognate XIP. As XIP peptide length may vary between ComRS systems, we synthesized and assessed two or three length variants for most species’ putative XIPs to determine the optimal peptide size needed to activate a given ComR. Each ComR tested exhibited P_*comS*_ reporter activity with at least one length-variant of the putative XIP (Fig 2D and S1 Fig).

Among all strains tested, the two Bovis representatives, strains SboB and 83, provided the most similar ComR alleles (82.6% identity) and XIP sequences (differing only in Ala or Ser at position 5 from the C-terminus, C-5). A roughly equal response to both *S*. *bovis* XIP-C7 variants was observed for both, and each displayed the lowest EC_50_ values recorded in our dataset (<<1 nM) (Fig 2C and 2D).

Among Pyogenic species, all five of the ComR proteins tested activated the cognate *comS* promoter, but to different maximal levels of activity and with variable sensitivities to XIP. Two Group B *Streptococcus* (GBS) ComRs (*Streptococcus agalactiae* 2603 v/r and A909) exhibited the strongest induction in reporter activity among the Pyogenics, with maximal activity observed with the C7 length of XIP. The ComRS alleles from Group A *Streptococcus* (GAS, *S*. *pyogenes*), MGAS5005 (a serotype M1 strain) and MGAS315 (serotype M3), showed slightly different activation profiles. The MGAS5005 ComR allele activated *P*_*comS*_::*luxAB* transcription at an EC50 value of approximately 250 nM with the C8 cognate XIP. However, upon treating the MGAS5005 reporter strain with 50 μM of the MGAS5005 C8 XIP, *P*_*comS*_::*luxAB* transcription increased approximately 25-fold while the MGAS315 C8 XIP elicited only 25% of the maximum transcription level at the same concentration (S1D Fig). One representative of the Lancefield G antigen group, *S*. *dysgalactiae* subsp. *equisimilis* strain ATCC35666, was included in our studies and responded best to the C7 cognate XIP, with an EC50 value of 15 μM and displayed ~10-fold increase in reporter activity at 50 μM XIP.

Additionally, we included *S*. *suis* in our analysis to expand the test-set to include more distantly related ComRS alleles. Unlike the type-I and type-II classifications, the *S*. *suis* XIP has a split aromatic motif with an acidic C-terminal side chain, in stark contrast to an aliphatic residue or glycine (Fig 1). However, ComR *S*. *suis* behaved similarly to the type-II ComRs in our transcriptional assay as it responded with the highest activity to the cognate C7 peptide with a EC_50_ of 93 nM (Fig 2C). Taken together, these results show that heterologous expression of *comR* alleles provides the capacity to implement pheromone responses, and they suggest that each *comRS* system provides quorum sensing functionality, albeit with potentially varying sensitivity, in their respective species.

### ComR variants exhibit disparate patterns of XIP selectivity

**Fig 3:**
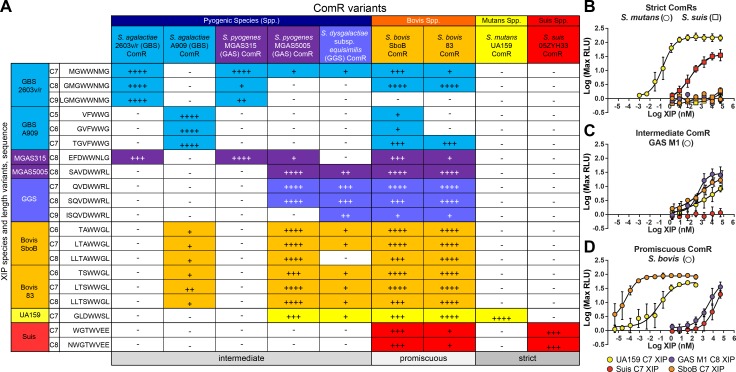
Type-II ComRs expressed in the ES1 (*ΔcomRS*) test-bed strain exposed to heterologous XIPs can be grouped into strict, intermediate or promiscuous ComRs based upon relative activity profiles. (A) Type-II ComR proteins were tested for the ability to promote luciferase expression using putative XIP proteins of varying length from both the same and heterologous species. The normalized test-bed activities for each ComR variant is defined as 100% for luminescence activity stimulated by the cognate XIP; activities from heterologous pairings are calculated by dividing the activity from the cognate XIP by the maximum luminescence attained from the heterologous XIP and represented as (++++) for >90%, (+++) for 70–89%, (++) for 50–69%, and (+) 25–49%, no observable induction is denoted by (-). Color indicates species. (B-D) Five representative strains that exhibit different ComR activity profiles: strict, intermediate, and promiscuous. (B) Strict ComRs, *S*. *mutans* UA159 (circles) and the *S*. *suis* 05ZYH33 (squares), exclusively respond to cognate XIPs, UA159 C7 XIP (yellow) and *suis* C7 (red), respectively. (C) The intermediate ComR of *S*. *pyogenes* MGAS5005 (circles) exhibits reporter activity when treated with the cognate GAS M1 C8 XIP (purple), UA159 C7 XIP (yellow), and the *S*. *bovis* SboB C7 XIP (orange) with different EC_50_ values. No activity is observed when treated with the *suis* C7 XIP (red). (D) The promiscuous ComR of *S*. *bovis* SboB (circles) exhibits strong activity upon treatment with the cognate SboB C7 XIP (orange) with an apparent EC_50_ of <1 nM. The SboB ComR also exhibits dose-responsive reporter activity upon treatment with the UA159 C7 XIP (yellow), the GAS M1 C8 XIP (purple) and Suis C7 XIP (red), which does not contain the WW-motif.

Although each XIP contains conserved aromatic residues, there is significant sequence variation of signaling peptides between species (Fig 1). Given this, we suspected that there might be structural aspects of the pheromones that denote specificity for their cognate ComR. To search for a possible pattern of substrate specificity, each ComR in our study was stimulated with non-cognate XIP peptides using the test-bed assay. Nine *comR* alleles were expressed in the *S*. *mutans* test bed strain, treated with titrations of each length-variant of XIP, and luciferase values were determined (S1 Fig). From these data, distinct activity patterns using heterologous XIPs were observed, leading to categorization of ComR proteins as “strict”, “intermediate”, and “promiscuous” (Fig 3).

The strict, or high-specificity group includes ComR alleles from *S*. *mutans* UA159 and *S*. *suis* 05ZYH33, both of which only exhibited detectable *P*_*comS*_ reporter activity upon addition of cognate XIP. The intermediate-specificity group included *S*. *pyogenes* MGAS315 and *S*. *agalactiae* strains 2603 and A909. These alleles displayed detectable reporter activities from one to three non-cognate XIP types. The promiscuous, or low-specificity group included ComR alleles capable of activation by a wide range of XIP variants, and included *S*. *pyogenes* MGAS5005, *S*. *dysgalactiae* subsp. *equisimilis*, and both *S*. *bovis* alleles. Each of these responded to at least five different XIP types, and *S*. *bovis* was found to have measurable responses to all eight other XIP peptides tested including the most disparate peptide derived from *S*. *suis* (Fig 3 and S1 Fig).

### X-ray crystal structure of ComR *S*. *suis*

**Fig 4:**
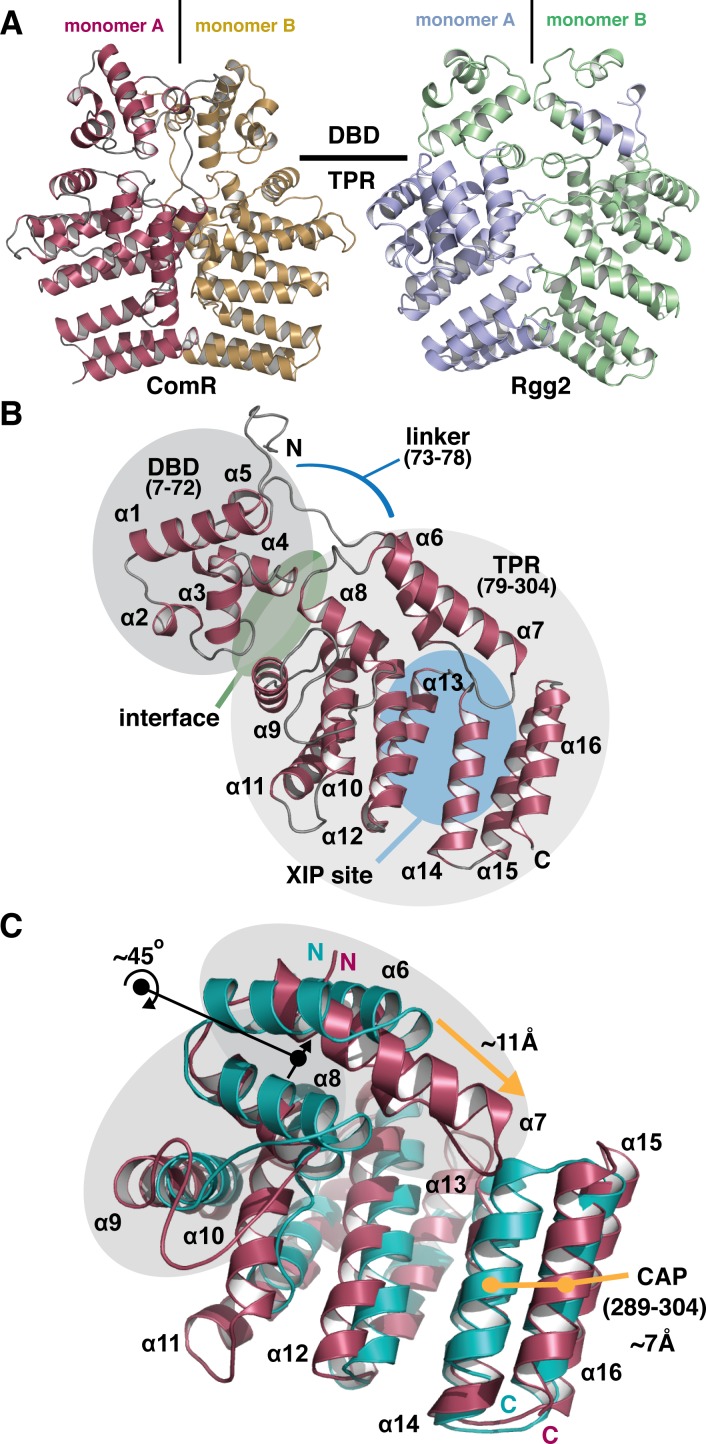
Overall structure of apo-ComR from *Streptococcus suis*. (A) Cartoon representation of the asymmetric unit of the native crystal form compared to the biological unit of Rgg2 (PDB code 4YV6). Each monomer is colored and the approximate boundary between the DNA binding domain (DBD) and the tetratricopeptide repeat domain (TPR) is indicated. (B) The biological unit of the apo-form of ComR. The DBD domain and TPR domains are shaded in grey with primary sequence boundaries listed in parenthesis with the extended loop that links the domains indicated by a blue line. The XIP binding region in the TPR is highlighted with a blue oval, in addition to the interface region between the DBD and TPR with a green oval. Each secondary structure element is labeled in by number order from N to C terminus (α = α-helix) (C) Alignment of the *S*. *suis* TRP (red) and Rgg2 TPR (teal). Black arrows and dots indicate a rotation of α-helices and orange arrows and dots show a translation of α-helices. The regions shaded in grey and the C-terminal CAP helix show significant differences.

To better understand the protein-pheromone interaction, we also pursued the structure of ComR. Although a structure for a type-II ComR proved elusive, ComR *S*. *suis* was of interest as it is part of the strict response group and recognizes a unique XIP. Furthermore, as ComR proteins are homologous, *S*. *suis* will provide a template for the accurate modeling of the other orthologs in our study. The native crystal structure of the apo-form of ComR *S*. *suis* reveals a dimerized α-helical protein in the asymmetric unit that resembles related Rgg-proteins [19] (Fig 4A, Table 1). However, the construct used in this study behaves mostly as a monomer in solution when assayed by size-exclusion chromatography (S2 Fig). As the active form of Rgg2 shows a domain swap of the DBD domains (Fig 4A) [19], the observed conformation of the ComR apo structure appears incompatible with DNA binding and would require reorientation of the DBD domains. This is reinforced by the observation that the DBD of ComR packs tightly against the TPR using α-helices 3 and 4 (DBD) and α-helices 8 and 9 (TPR) to form a significant interface region (Fig 4B). The observed dimer interface between the TPR domains appears to help promote crystal-packing in this structure and the two monomers exhibit an identical conformation (RMSD = 0.192 Å^2^). The monomer is shown in Fig 4B, revealing that ComR *S*. *suis* is comprised of 16 α-helices divided into two domains. Helices 1–5 (residues 7–72) constitute the highly conserved DNA-binding-domain (DBD) and helices 6–16 (residues 79–304) comprise the tetratricopeptide repeat (TPR) domain that is the predicted site of interaction with XIP. These two domains are separated by a 6 aa linker region. It is also important to note that the space group of the selenomethionine derivative compared to the native crystal resulted in a different packing arrangement with only one monomer per asymmetric unit versus two monomers for the native crystal. Due to these packing differences, clear electron density for α-helices 15 and 16 of the TPR domain is only visible in the native data set (S3 Fig). Furthermore, the derivative had other areas of discontinuous density leading to an incomplete model and a very poor overall refinement. As several areas of weak density leaves doubts about the model and the conformations of α-helices 15 and 16, the derivative structure has been omitted despite the slightly better resolution of the dataset.

**Table 1:** X-ray crystallographic data collection and refinement statistics

	Native	SeMet
**Data Collection**		
Space group	P3_1_21	P3_2_21
Cell dimensions		
a, b, c (Å)	82.3, 82.3, 209.8	75.3, 75.3, 96.4
α,β,γ (°)	90.0, 90.0, 120.0	90.0, 90.0, 120.0
Wavelength	0.97851	0.97872
Resolution (Å)	52.0–2.9	30–2.7
R_meas_	8.9 (126.8)	5.7 (174.2)
CC(1/2)	99.9 (75.2)	99.4 (48.4)
*I/σI*	12.3 (1.2)	16.3 (0.9)
Completeness (%)	99.3 (98.7)	98.2 (91.1)
Redundancy	5.9 (6.0)	4.8 (4.6)
**Refinement**		
Resolution (Å)	29.5–2.9	
Number of monomers in asymmetric unit	2	
R_work_/R_free_	22.0/25.5	
No. atoms		
Protein	5160	
*B*-factors		
Protein	110.0	
R.m.s deviations		
Bond lengths (Å)	0.008	
Bond angles (°)	1.119	
Ramachandran plot (%)		
Favored	97.2	
Allowed	2.5	
Outliers	0.3	

Although this general architecture of the monomer is observed in a related Rgg protein [19] and other RNPP family members such as PlcR [17, 19, 27], ComR *S*. *suis* differs from them in several ways, foremost in that the apo-form of the protein in solution is a monomer, not a dimer (Fig 4 and S2 Fig). ComR *S*. *suis* also lacks the disulfide bond observed in the DBD of Rgg2 that is thought to help facilitate its stable dimeric assembly and orient these domains for interaction with DNA [19]. Furthermore, a comparison of the ComR TPR to the Rgg2 TPR reveals several differences (Fig 4C), indicating that the activation mechanism likely differs significantly from Rgg proteins. Overall, the size and shape of the ligand binding pocket is altered due to a re-arrangement of several of the helices. Notably, α7 extends into the putative peptide binding site and helices α6–8 are rotated by 45° relative to Rgg2. This and the additional length of α7 in ComR allows for an ~11Å extension to contact helices α14 and α16. Finally, the C-terminal capping helix (CAP) [19] of ComR *S*. *suis* is translated outward by ~7Å relative to Rgg2. These differences may determine substrate specificity. For example, the configuration of ComR would block the binding of Cyclosporin A, an inhibitor of Rgg2 [19, 28].

### Analysis of the molecular surface of ComR

**Fig 5:**
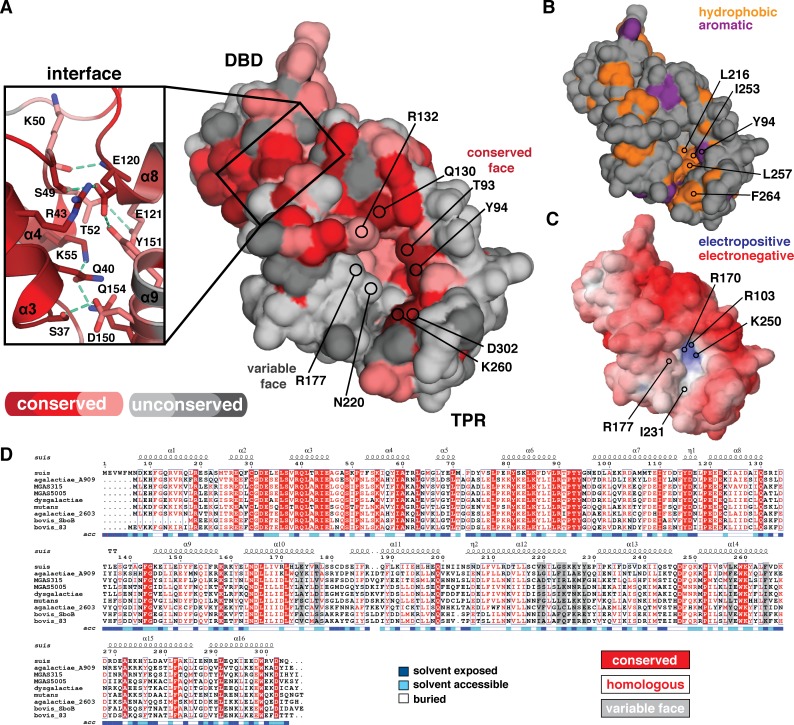
The molecular surface properties of apo-ComR *S*. *suis*. (A) Solvent accessible surface representation colored by amino-acid conservation using the Consurf server and the bacterial strains used in this study. Darker red indicates increased conservation with dark red showing completely conserved positions. Unconserved residues are shown in grey, with dark grey as least conserved. The XIP binding site consists of a conserved surface and a variable surface. Residues of interest are indicated by position and labeled. The inset shows the DBD and TPR domain interface that consists of highly conserved residues, with hydrogen bonds indicated by a dashed green line. (B) The solvent accessible hydrophobic residues of the ComR *S*. *suis* surface are highlighted in orange (C) Electrostatic surface potential as calculated by APBS with a contour of -10 kT/e to 10 kT/e. (D) Alignment of ComR *S*. *suis* with all species studied in this work. Secondary structure is annotated as Fig 4. Conserved and homologous residues are highlighted in red with the XIP variable face residues in a gray box. The alignment was generated by Clustal Omega and Espript3

To understand how ComR *S*. *suis* recognizes its peptide and how ComR proteins generally may discriminate or accommodate heterologous XIP substrates, the molecular surface of the apo conformation of ComR was examined in detail (Fig 5). ComR from strains used in this study were aligned with Clustal Omega [29] and submitted to the Consurf server [30, 31] to plot areas of sequence conservation. As shown in Fig 5A and 5D, although the DBD is highly conserved, the TPRs diverge significantly so that only one side of the putative XIP binding-site pocket exhibits sequence conservation among the ComR species in this study. This creates a “conserved face” and “variable face” on opposing surfaces of the cavity for potential interaction with peptide pheromones. Residues that show strict conservation line the outer edge of one side of the pocket, whereas the inside of the pocket shows some minor variation. The variable face likewise lines one-half of the binding surface and includes a deep hydrophobic groove in the unbound state of the pocket, at least in *S*. *suis* (Fig 5A and 5B). Based on this observation we hypothesize that the conserved face of the XIP interface contains elements required for peptide binding and that the variable face acts in substrate specificity. However, these activities are likely not mutually exclusive, as suggested by the electrostatic potential of ComR *S*. *suis* generated by the Adaptive Poisson-Boltzman Solver software (APBS) [32, 33] (Fig 5C). The homologous residues of the inner pocket are electropositive (R103, R170, and K250) which is likely required for optimal interaction with the C-terminal glutamic acid moiety of the ComR *S*. *suis* XIP (Fig 1).

When considering general residue conservation, the DBD-TPR domain interface is also a site of interest (Fig 5A inlay). All residues at the domain interface are conserved or homologous, polar, and create an extensive network of hydrogen bonds and salt-bridges that stabilize the observed conformation, showing that this is not a crystal-packing artifact. Indeed, relative to Rgg2 the DBD of *S*. *suis* appears to be held in an inactive conformation to occlude helices α3 and α4 from interacting with DNA and possibly inhibiting dimerization.

Given the specificity of various ComRs to heterologous XIPs (S1 Fig), we also performed a molecular surface comparison between strict and promiscuous ComR proteins to look for common or differing structural features that may correlate to these activities. All proteins used in this study were modeled using the program Modeller with apo-ComR *S*. *suis* as a template [34] and their surface properties explored, with four representative structures selected to represent the analysis (*S*. *suis*, *S*. *mutans*, MGAS5005, and *S*. *bovis 83*) (S4 Fig). We observe that strict ComR proteins appear to have a significantly more electropositive pocket than ComRs that can recognize multiple pheromones (S4A Fig), but that the overall hydrophobicity of the pocket does not seem to differ between recognition types (S4B Fig). We examined the size and shape of the apo-pocket using the 3V webserver [35], which suggests that the promiscuous ComR binding pockets maybe smaller and shallower, with slightly more aliphatic character (S4C Fig). However, it is important to note that the difference in pocket size is not extremely pronounced and thus likely falls within the range of the error of the homology model.

### Structural analysis of the pheromone binding pocket of ComR provides clues to peptide selectivity

**Fig 6:**
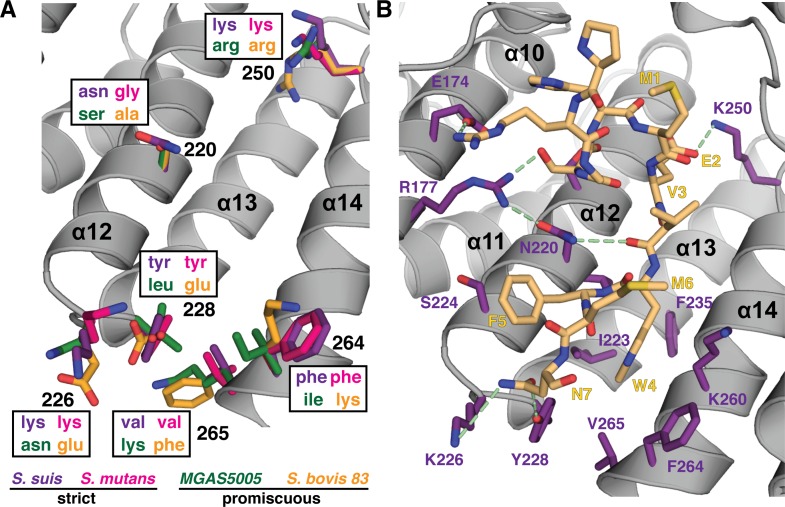
Structural comparison of the XIP binding pocket between different peptide recognition types. (A) Residues from strict and promiscuous ComR proteins are compared and labeled by color. *S*. *suis* in purple, *S*. *mutans* in pink, *MGAS5005* in green, and *S*. *bovis* 83 in orange. (B) Crystal packing artifact of the N-terminus (yellow) may mimic initial ComR peptide recognition. Residues making van der Waals contacts and/or hydrogen bond with the peptide are shown in purple, hydrogen bonds are represented by a dashed green line.

As we observed possible trends in the overall molecular features of the pheromone binding site, we examined the variable face for specific features that may be common to strict or promiscuous ComR proteins. As shown in Fig 6A, there is a residue consensus for the strict ComR proteins that occurs at the lower end of the pocket. Positions 226, 228, 264 and 265 are identical between *S*. *suis* and *S*. *mutans*, but variable in the promiscuous examples. This is in contrast to residues in the middle of the variable face, which are unconserved (Fig 5A). However, residues on the conserved face do show some variability, although they are homologous and preserve the general side chain characteristics. For example, both strict ComR proteins have lysine at position 250 as opposed to arginine for the promiscuous examples (Fig 6A), but it is unlikely that this subtle difference affects peptide binding.

Recent work comparing RNPP family structures has shown that the presence of an Asn residue in the XIP binding pocket appears to be a common feature [36], however it is not clear if this is true for ComR proteins. No residue on the conserved face is an asparagine (Fig 5), and although ComR *S*. *thermophilus* uses N208 to contact XIP (see Talagas. et. al. [37]), that same position in ComR *S*. *suis* is D213 (S5A Fig). Given that an aspartic acid likely fills a homologous role, it is tempting to speculate that variation of the position of an Asn residue may be related to peptide specificity. However, it is not readily apparent if the position an Asn relates to our observed categories of responses to XIP, for example (Fig 3 and S5B and S5C Fig). Furthermore, an alignment of the ComR X-ray structures and models used in this study reveals that MGAS5005 may not have an Asn or Asp in a position for contact with XIP (Fig 5 and S5C Fig).

**Fig 7:**
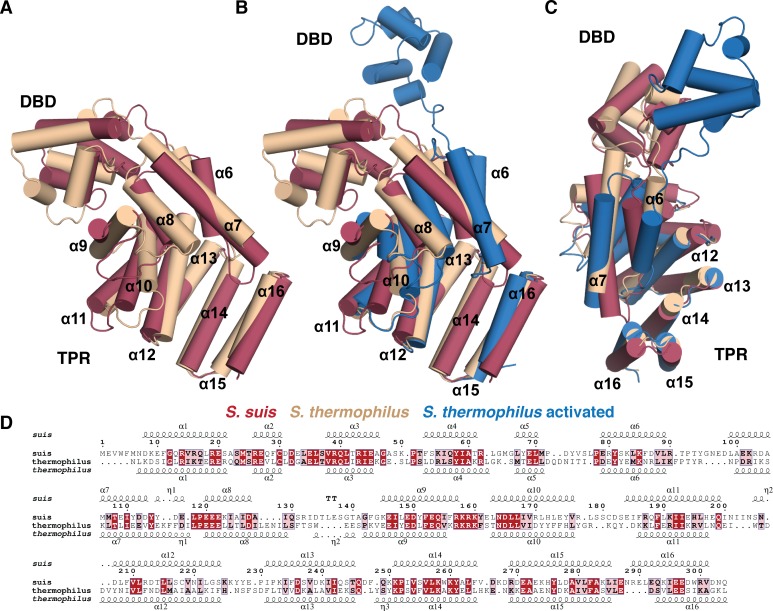
Structural comparison of ComR *S*. *suis* and ComR *S*. *thermophilus*. Alignment of ComR monomers using the TPR domain. (A) Alignment of the two apo-structures, (B) as (A) including the monomer from the ComR-ComS-DNA ternary complex. (C) Is panel (B) looking at the TPR from the activated DBD conformation (rotated y, -90° and x, 45° from the middle panel) (D) Sequence conservation and homology between ComR *S*. *suis* and *S*. *thermophilus*. Conserved residues are boxed in red with white lettering and homologous residues in pink with black lettering. The DNA binding domain (DBD) and TPR are labeled and helices labeled by number. The alignment was generated by PDBeFold and Espript3.

Although the features highlighted in Fig 6A show a trend, in the type-I ComR-XIP *S*. *thermophilus* structure (Talagas, *et al*. [37]) the lower end of the pocket does not interact with the bound peptide, including residue K260 on the conserved face that is strictly conserved in all ComR strains used in our study (Fig 5, Fig 6B, S5A and S5B Fig). This could suggest a different mode of interaction with XIP, possibly in a more extended conformation, or that K260 performs an accessory role such as help to orient the CAP helix given that it forms a salt-bridge with D302 (Fig 6). However, our crystal structure contains a crystal-packing artifact that not only hints at how ComR *S*. *suis* recognizes its unique XIP, but provides clues for initial peptide recognition or selection. As shown in Fig 6B, the N-terminal residues (1 to 7 plus 4 residues of the cloning linker, RGSH-MEVWFMN) from a symmetry-related ComR bind in the apo-conformation of the XIP pocket. Although bound in the reverse orientation to *S*. *thermophilus* (S5 Fig), several hydrogen-bonds and van der Waals contacts are made to both the conserved and variable face with an interaction surface area of 1269 Å^2^ for the entire contact, as determined by PISA (http://www.ebi.ac.uk/pdbe/prot_int/pistart.html) [38]. Notably, W4 slots into the deep hydrophobic pocket of the variable face (Fig 5 and Fig 6B) while packing against K260, and E2 forms a salt-bridge with K250 at the back of the pocket, possibly mimicking the side-chain interactions of the *S*. *suis* C7 peptide. Additionally, residues that are common to strict ComR proteins (Fig 6A) hydrogen-bond to the N7 side-chain (residues K226 and Y228) while the variable face residue N220 is involved in hydrogen-bonding to the peptide backbone of the artifact. It is also important to note that this peptide fails to make contacts with residues T93 and R103 on α7 (T90 and K100 in *S*. *thermophilus*), which appear crucial to inducing the conformational change of the TPR necessary for activation (S5A and S5B Fig). Given this, we hypothesize that this may mimic in part an initial peptide interaction event that occurs when the XIP binding pocket is in the apo conformation. If the interacting XIP is accepted it can then trigger the conformational change of the TPR domain to open the DBD-TPR interface to allow dimer formation and activation as proposed by Talagas, et al. (Fig 7 and S5 Fig) [37].

### The ComR *S*. *suis* and ComR *S*. *thermophilus* TPR domain have distinct structural features

To further explore the molecular features of the XIP binding pocket, we also compared the type-III ComR structure directly to the type-I ComR apo and activated structures reported in Talagas, et. al. (Fig 7 and S5 Fig) [37]. As shown in Fig 7A, the conformation of the TPR domain of *S*. *suis* and apo *S*. *thermophilus* structure are similar, although there are noticeable differences. Helices α6, α9, and α10 adopt clearly distinct conformations, and *S*. *suis* α7 extends further over the XIP binding pocket to contact the CAP helix α16. This arrangement is perhaps in part responsible for the observed conformational difference of the DBD domain relative to the TPR. Furthermore, the loop region between α8 and α9 (residues 128–144 *S*. *suis* and 131–139 *S*. *thermophilus*) is longer in *S*. *suis*, and the loop connecting α10-α11 (residues 178–183 *S*. *suis* and 173–181 *S*. *thermophilus*) adopts a different conformation between the two structures. In *S*. *thermophilus* this loop folds facing into the XIP pocket, but in *S*. *suis* this loop faces out towards the solvent (Fig 7A and S5A Fig).

Interestingly, if we compare these structural features to the activated ternary complex (Fig 7B) we observe that the XIP binding pocket of apo-ComR *S*. *thermophilus* shares more in common with the activated complex than the ComR *S*. *suis* structure (Fig 7C). Helices α10, α12, and α16 of *S*. *suis* differ from the *S*. *thermophilus* structures and the general position of the α10-α11 loop that makes contacts with XIP is preserved in both ComR structures of *S*. *thermophilus* regardless of whether the peptide is bound (Fig 7B and S5A Fig). In the activated state, the TPR as a whole does undergo significant changes most notably at α9 and α10 (including the loop region) and the repositioning of α6-α8 through contact with XIP. When activated, α6-α8 appear to rotate by ~30° relative to the apo-conformation (Fig 7B). However, we see that α6 and α7 in *S*. *suis* exhibit a different conformation from both type-I structures as they appear to be rotated counter clockwise (Fig 7C). Given that the type-I and type-III ComR proteins studied here recognize significantly different peptides (Fig 1) and have insertions/deletions of their primary sequence, these differences could potentially correlate to how XIP is recognized. However, as the DBD is inherently dynamic and the observed crystal packing interactions include some of the dimer contacts (Fig 4A), we cannot rule out influence from the crystallization process on the observed conformations of the *S*. *suis* or *S*. *thermophilus* structures.

### Contributions of conserved and variable faces to the recognition of XIP

**Fig 8:**
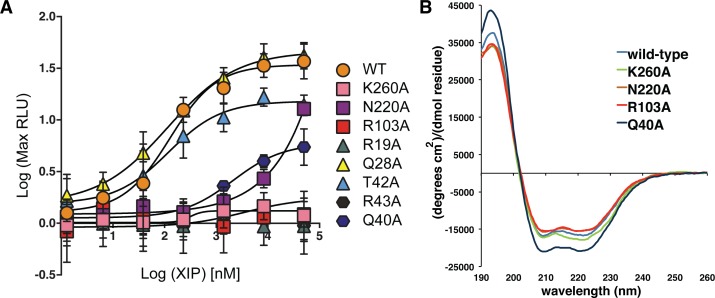
Luciferase reporter assay results of ComR *S*. *suis* mutants stimulated with Suis C7 XIP. **(A)** Point-mutations were introduced into ComR *S*. *suis* in the DBD (triangles R19A, Q28A, T42A), the DBD-TPR interface (hexagons Q40A, R43A), or in the conserved (K260A, R103A) or variable surfaces (N220A) of the XIP binding pocket (square symbols). Wild-type *S*. *suis* ComR activity with Suis C7 XIP is depicted as circles. **(B)** Circular dichroism spectra of purified variant proteins of interest displaying decreased transcriptional activity compared to wild-type, plotted as mean residue ellipticity.

As we have hypothesized that specific contributions of the conserved and variable faces participate in binding the peptide pheromone, and that ComR discriminates between, or filters, XIPs with the apo conformation of the pocket including residues in the lower end of the putative interface, we tested the interaction of ComR *S*. *suis* with peptide by site-directed mutagenesis. We created several protein variants of ComR *S*. *suis* to probe the assorted molecular features, including the solvent-exposed surface of the DBD (R19A, Q28A, and T42A), the DBD-TPR interface (Q40A and R43A), a residue on the conserved surface and lower end of the pocket (K260A), the electropositive surface of the back of the pocket (R103A, K100 in *S*. *thermophilus* see Talagas et. al. [37]), and the variable face (N220A) (Fig 5, S5B Fig and Fig 8A). These variants were placed into the ES1 test-bed background, and luciferase activity was recorded after stimulation with the *S*. *suis* XIP-C7 as outlined in Fig 2A. Additionally as a control, several variants were also introduced into the expression plasmid, purified as the wild-type, and assayed for structural integrity by circular dichroism (CD) spectroscopy (Fig 8B). The spectrum of each variant tested shows a folded α-helical protein similar to wild-type ComR *S*. *suis*, indicating that the selected mutations did not disrupt the protein fold.

The DBD variants Q28A and T42A retained significant activity, with Q28A responding to XIP similarly to wild-type. ComR *S*. *suis* R19A showed no activity and likely contributes indirectly through contacts made to residues on α-helix 3 that make close contacts with DNA (Fig 8A) [19]. Both residues at the DBD-TPR interface (Q40A and R43A) also exhibited significantly reduced activity, although Q40A could be partially stimulated with high levels of XIP. As Q40A and R43 are on α-helix 3, a conformational change would be required for interaction with DNA (Fig 5A, Fig 7, and Talagas et. al. [37]). The loss in activity could result from either disruption of the regulator mechanism or reduced affinity for DNA. In the case of the conserved residue K260 of the lower pocket, when it is mutated to alanine only negligible activity was observed, indicating that it is required for the activation of ComR. The same effect is observed for R103, implying that proper electrostatic interactions with the *S*. *suis* XIP are necessary. Interestingly, we also observed that mutation of residue N220 severely reduced activation, demonstrating that the variable face of ComR *S*. *suis* participates directly in the activation of ComR.

**Fig 9:**
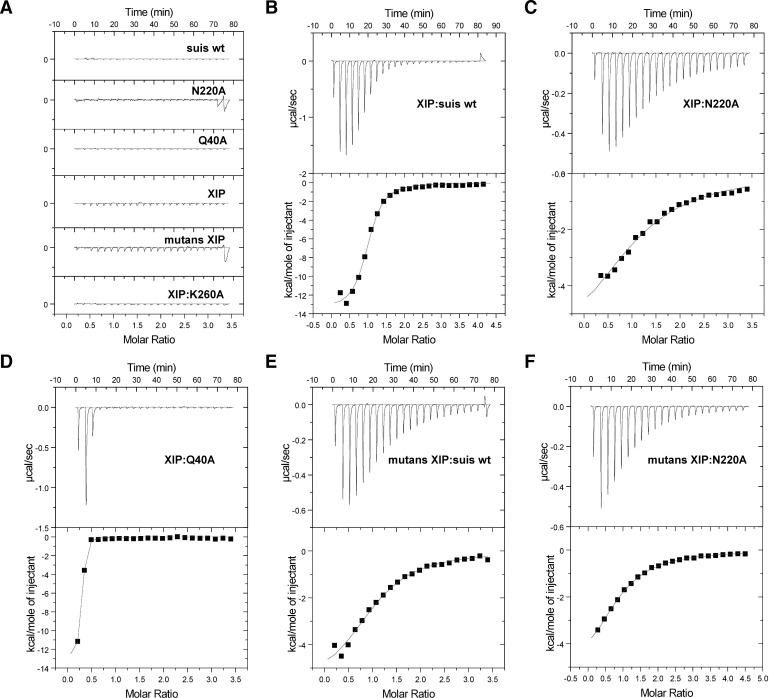
Isothermal titration calorimetry of ComR variants and XIP. (A) Heats from control titrations are shown. Buffer was titrated into wild-type ComR *S*. *suis* and each variant protein, whereas XIP was titrated into buffer. The bottom panel shows the titration of *S*. *suis* XIP-C7 and the conserved surface variant K260A. (B) Titration curve for *S*. *suis* wild-type and cognate XIP-C7. (C) Titration of the variable face variant N220A with cognate XIP C7. (D) Titration of DBD and TPR interface variant Q40A with cognate XIP-C7. (E) Titration of *S*. *suis* wild-type with *S*. *mutans* XIP C7. (F) Titration of the variable face variant N220A with *S*. *mutans* XIP C7. Binding constants and thermodynamic values are presented in Table 2.

We next sought to examine further the specific mechanism of how ComR recognizes XIP directly *in vitro* through isothermal titration calorimetry (ITC) using three of the purified variants in Fig 8B. The titration curves of each experiment are shown in Fig 9 and the results summarized in Table 2. As shown in Fig 9B, *S*. *suis* has a high affinity for its cognate XIP-C7 with a K_d_ similar to that observed for SHPs and Rggs (Table 2) [39]. Additionally, the binding mechanism is driven by hydrogen bonding and likely electrostatic interactions as indicated by the large enthalpy, which again emphasizes the importance of the electropositive pocket (Fig 5C). When K260A was titrated with peptide (Fig 9A) we observed only background heats, indicating that K260A is absolutely required for binding of XIP. Mutation of N220 in the variable face of the pocket reduced the affinity for peptide by more than an order of magnitude (0.86 μM to 13.7 μM), although the overall mechanism appears to be similar (Table 2, the sign for ΔH and ΔS is the same as wild-type). Oddly, titration of XIP with the DBD-TPR interface variant Q40A showed rapid saturation suggesting an altered interaction with XIP or aggregation of the protein (Fig 9D). As Q40 may be part of the regulation mechanism, the protein variants that could still bind XIP were also examined by dynamic light scattering (DLS) (S6 Fig). The purified wild-type ComR *S*. *suis* showed one species in solution and then conversion to a larger species in the presence of XIP (S6A and S6B Fig). Likewise, N220A had the same behavior in solution as wild-type in the absence of XIP (S6C Fig). However, Q40A on its own appeared to have already dimerized and was in equilibrium with larger aggregates at a ratio of 4 to 1 (S6D Fig). Given that the DBD-TPR interface is likely compromised in this variant, the loss of activity of Q40A appears to be due to dis-regulation of dimer formation, which results in a significant amount of non-specific unproductive oligomerization. In this context, due to the observed aggregation by DLS, the interaction of XIP with Q40A remains unclear.

**Table 2:** Isothermal titration calorimetry of ComR *S*. *suis* variants and XIP

ComR	XIP	N	(+/-)	K_d_ (μM)	(+/-)	ΔH (cal mol^-1^)	(+/-)	ΔS (cal K^-1^ mol ^-1^)
wild-type	suis C7	0.96	0.015	0.862	0.121	-13550	319	-17.76
K260A	suis C7	-	-	-	-	-	-	-
N220A	suis C7	1.092	0.139	13.7	3	-7280	1228	-2.186
wild-type	mutans C7	1.035	0.05	5.88	0.968	-6013	408	3.75
N220A	mutans C7	0.95	0.069	6.06	1.29	-4259	413	9.58

As we were interested in the binding of heterologous XIPs by ComR (S1 Fig and Fig 3), ComR *S*. *suis* was also titrated with *S*. *mutans* XIP C7 (Fig 9E). Although ComR *S*. *suis* cannot be activated by *S*. *mutans* XIP (Fig 3 and S1 Fig), it is still able to bind the pheromone directly as observed by ITC. However, the affinity is reduced by 7-fold and the mechanism of binding has changed, most notably a positive contribution from entropy (Table 2). Additionally, we titrated N220A with *S*. *mutans* XIP and observed the same K_d_ and mechanism as wild-type ComR *S*. *suis* (Fig 9F, Table 2). Together these data support our hypothesis that the conserved face residues provide the required contacts for pheromone binding while the variable face provides XIP-selectivity.

### Prediction and design of synthetic XIP agonists

**Fig 10:**
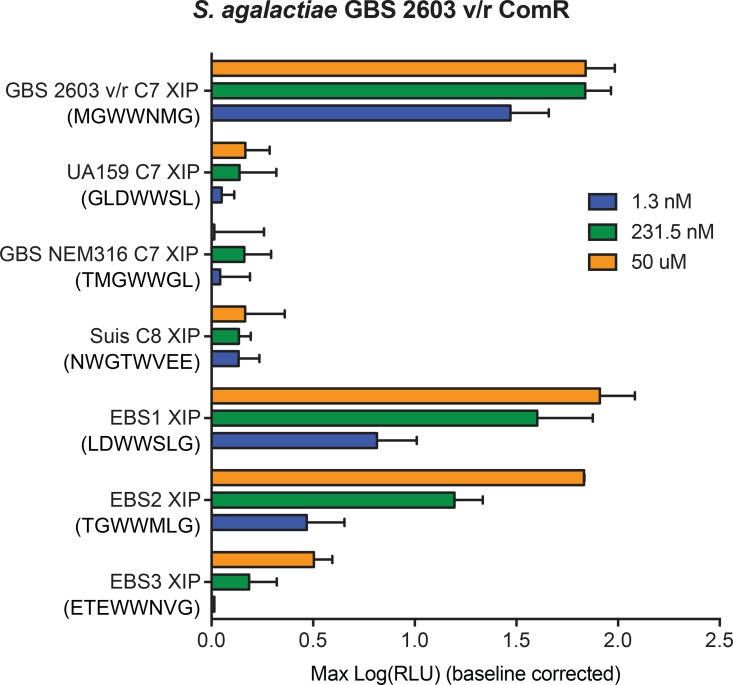
Designed XIP agonists of ComR *S*. *agalactiae* 2603. XIP peptides found to be ineffective signals for ComR *S*. *agalactiae* 2603 were redesigned to satisfy hypothetical ComR-activating criteria. Designed synthetic peptides were titrated to strain ES1 carrying ComR *S*. *agalactie 2603* reporter and maximum relative luciferase activities were recorded.

Observed pheromone specificity patterns (S1 Fig and Fig 3) and clues revealed from the ComR structure (Fig 5) suggested it might be possible to activate a selective ComR variant using a non-inducing XIP peptide redesigned to satisfy criteria for activation. For these experiments, the intermediately-restrictive *S*. *agalactiae* 2603 ComR was chosen since two disparate XIP peptides displayed activity upon this ComR, offering a basis for activation criteria. By comparing common traits of active peptides to those of inactive peptides, we identified two primary characteristics we hypothesized would account for activity. First, all peptides active with ComR *S*. *agalactiae* 2603 have three residues after the WW-motif, whereas all inactive peptides have one or two residues. Second, active peptides typically contain a terminal glycine or hydrophobic residue. A chemically-compatible terminal residue appears to be critical as the structure of the *S*. *thermophilus* ComR-XIP complex shows that it is inserted into the back of the XIP pocket to induce dimer formation (Talagas, et al. [37]). This is also observed in *S*. *suis* where the positive charge of the pocket and negative charge of the XIP play a role in binding (Fig 5 and Fig 7). Using these observations as a basis for ComR *S*. *agalactiae* 2603 activity requirements, we chose three XIP variants from *S*. *mutans*, *S*. *suis* and different *S*. *agalactiae* strain (NEM316, XIP TMGWWGL) that were unable to activate ComR *S*. *agalactiae* 2603 and predicted sequence rearrangements of the peptides that would lead to ComR activation. Each peptide was rearranged to fit the guidelines for ComR *S*. *agalactiae* 2603 to include three residues after the adjacent WW motif and contain a C-terminal glycine, leading to EBS1 (LDWWSLG), EBS2 (TGWWMLG), and EBS3 (ETEWWNVG). When tested by titrating the synthetic peptides to the ES1 reporter strain carrying ComR *S*. *agalactiae* 2603, EBS1 and EBS2 exhibited robust reporter induction (Fig 10) indicating that the guidelines successfully generated novel XIP ligands in two of three instances. However, the EBS3 XIP, which contains eight residues (rather than seven in EBS1 and ESB2) did not elicit strong reporter activity, indicating that pheromone length is an important determinant of activation, as reported in ComR *S*. *thermophilus*.

## Discussion

Through efforts to understand in detail the species specificity and molecular mechanism of ComRS quorum sensing, we developed an *in vivo* technology to test and monitor *Streptococcus spp*. communication in the regulation of competence. In prior studies with *S*. *mutans* and *S*. *thermophilus*, the exogenous addition of synthetic XIP was sufficient to activate ComR and induce transformation when cultures were grown in a chemically defined medium (CDM [10, 22, 40]). These discoveries enhanced the ability to genetically manipulate naturally transformable species under laboratory conditions [41, 42]. Nonetheless, for many notable pathogens, including *Streptococcus pyogenes*, exogenous addition of XIP leads to SigX induction, but transformation under laboratory conditions remains elusive [14]. The use of the *S*. *mutans* ‘test bed’ not only opens up the study of new ComRS systems, but has shown that ComR proteins can be strict or promiscuous in their ability to recognize the QS messengers of other species. We expanded these results by determining an X-ray crystal structure of ComR *S*. *suis* to help explain observations of pheromone specificity and develop a model for initial peptide recognition and activation of ComR. The biochemical data show that the XIP binding pocket consists of two faces, a conserved face that is required for peptide binding, and a variable face that we propose functions in pheromone specificity. Furthermore, the structural and biochemical observations together strongly suggest that the observed apo conformation of the XIP binding pocket allows the binding of varied XIP sequences. However, only those that satisfy the correct molecular interactions with both the conserved and variable face with sufficient affinity can induce the conformational change for dimerization that is required to bind DNA.

Genetic manipulation of many important pathogenic streptococcal species has been notoriously tedious, making the study of several species prohibitively difficult [43]. Here we demonstrate the expression of heterologous genes in an *S*. *mutans* test bed as a way to bypass slow and arduous genetic manipulation of native species to study genetic pathways. Through exchange of coding and regulatory regions of *comRS* loci from various species, we determined the functionality of ComR/XIP pairings within an otherwise intact quorum-sensing system. Our results indicate that nine studied systems exhibited productive pairings between pheromone and receptor, providing further evidence that the ComRS QS pathway is functionally maintained for the induction of the SigX regulon across Suis, Bovis, Mutans and Pyogenic species of *Streptococcus* [10, 14, 41, 42]. These findings demonstrate that previously unstudied ComRs from *S*. *dysgalactiae* and *S*. *agalactiae* bind the cognate XIP *in vivo* to activate transcription. Furthermore, the use of *S*. *mutans* as a host system could be easily translated to the study of other RNPP proteins and encoded peptide ligands found among the order *Lactobacillales*, or potentially be adapted to study the activity of other gene pairs. Taking into account the significant level of sequence variability of ComR and ComS peptides, we used this test-bed method to assess the ability of different ComR proteins to recognize foreign XIPs. We selected species from the Pyogenic (GAS and GBS), Bovis, and Mutans groups, and included the peculiar example of *S*. *suis* due to its distinctive split-tryptophan motif in ComS, which allowed us to discover unique response profiles that ranged from highly-specific to highly-promiscuous.

The two most promiscuous ComRs included in our study, SboB and Sbo 83, both recognized peptides encoded by species of each group within the type-II ComRS family. Surprisingly, both ComRs also recognized the atypical XIP encoded by *S*. *suis*. Together, these results suggest that the Bovis peptide-binding pocket’s intrinsic properties allow for more permissive ligand binding. Recently, Morrison *et al*. demonstrated that natural transformation in *S*. *infantarius* is activated under laboratory conditions upon treatment with synthetic cognate XIP [41]. Our results suggest, assuming successful import via Opp, that all type-II and type-III *S*. *suis* derived XIPs can activate competence in this Bovis species.

The results presented here agree with previous findings that ComR and putative XIPs up-regulate the competence regulon in *S*. *infantarius*, *S*. *suis*, and *S*. *pyogenes*, [14, 41, 42]; however, modest differences with published findings were seen with the test-bed system. One observed difference from published ComR/XIP behavior was for the two *S*. *pyogenes* strains used in our study, MGAS5005 (serotype M1) and MGAS315 (serotype M3). In the ES1 background, both the MGAS5005 and MGAS315 ComRs exhibited specificity with respect to the M1 and M3 versions of XIP, contrary to the robust cross-strain activation of *P*_*sigX*_::*luxAB* between the GAS M1 and M3 ComRS systems observed by Mashburn-Warren [14]. Though we are unable to explain these discrepancies, it is possible that M1 and M3 XIPs display differential stability within *S*. *mutans* extracellular or intracellular environments, possibly due to expressed peptidases that degrade peptide signals seen in other systems [44, 45]. Another observed difference is in comparison to past work showing that *S*. *suis* S10 (serotype 2) had the highest transformability upon addition of the XIP-C9 length variant (GNWGTWVEE), and that the C8 variant (NWGTWVEE) activated higher levels of transformation than that of the C7 variant [42]. Our findings indicate that an *S*. *suis* ComR of a different strain (05ZYH33) but same serotype expressed in the *S*. *mutans* ES1 background more strongly activated *P*_*comS*_::*luxAB* upon treatment with the C7 length (WGTWVEE) than with C8. As both Suis strains encode identical ComR and ComS genes, this difference in activity might be explained by a length preference of the *S*. *mutans* peptide permease Opp. Substrate selectivity of peptide transporters has been reported, as in studies with *Lactococcus lactis*, where Detmers *et al*. observed a significant decrease in affinity between OppA and peptide substrates when the peptides contained 8 or 10 amino acids instead of the preferred 9, regardless of amino acid identities [46].

In comparing the biochemical features of ComR with other Rgg-like proteins, we found that unlike Rgg2, apo-ComR is a mostly a monomer in solution (S2 Fig) [19]. Previous work demonstrated that the active ComR-XIP complex is a dimer [21], and our DLS results demonstrate dimerization *in vitro* upon the addition of XIP (S6 Fig). However, the asymmetric unit of the *S*. *suis* ComR crystal structure resembles that of dimeric Rgg2, save for the lack the DBD domain swap (Fig 4A). The DBD is instead packed against the TPR by a substantial hydrogen-bonding network, which includes several residues implicated in DNA binding (Fig 4B, Fig 5A). Interestingly, when the residues at the domain interface are mutated, activity is abolished as determined by transcriptional reporter (Q40A, R43A Fig 8). Moreover, if the hydrogen-bonding stabilization of the DBD-TPR interface is disrupted, apo-ComR forms dimers and higher order oligomers, at least *in vitro*, that likely interfere with proper activity (Fig 5 and S6 Fig). From this we conclude that the apo-ComR structure holds the DBD against the TPR to not only time the DBD domain swap with XIP binding, but to assure proper oligomerization for the interaction with DNA. These observations are in complete agreement with Talagas et. al. [37] suggesting that the proposed dimerization mechanism is persevered in type-I, type-II, and type-III ComR proteins.

Due to the activation mechanism of ComR, there are several additional significant structural differences from other Rgg proteins. Not only does ComR lack the Rgg2 DBD disulfide to force oligomerization [19], but also the pheromone binding pocket itself differs drastically. As shown in Fig 4C, the arrangement of the ComR TPR produces a significantly different pocket. The most important feature is the extended helix α7 that makes contacts with the ComR CAP helix. We see a large shift of the ComR CAP outwards which not only produces a deeper apo-pocket compared to Rgg2 but the CAP-α7 interaction is likely broken by XIP binding, which in turn would allow for a shifting of the α6-7-8 helix bundle to begin the release of the DBD for dimerization. This suggests a completely different mode of peptide binding and activation of ComR compared to Rgg proteins and other members of the RNPP family.

ComR-XIP interaction results from the test bed experiments led us to classify ComR proteins as strict, intermediate, and promiscuous (S1 Fig and Fig 3). Regardless, a general comparison of the molecular features of the XIP binding pocket between these types did not reveal outstanding molecular differences to account for specificity (S4 Fig). Although some qualitative differences appear to exist between strict ComRs and promiscuous ComR TPR domains, these features did not correlate directly to complementary XIP charge or XIP side-chain size. Instead we observe that the ability to be activated by XIP depends on key interactions with the pocket side-chains themselves, likely in a species-specific manner. Supporting this hypothesis, we have observed that the apo-ComR XIP binding pocket consists of a conserved face and a variable face (Fig 5).

The amino acid conservation map of the apo-ComR surface in Fig 5 demonstrates one half of the pocket to be conserved and the other variable. As expected, if the conserved residues are mutated transcription is abolished (R103A, K260A Fig 8A) due to the inability of XIP to bind ComR (K260A Fig 9A). Moreover, K260 does not contact the activated peptide conformation as shown in Talagas et. al. [37] and S5 Fig. This suggests either a different conformation for the *S*. *suis* XIP, or that the lower end of the pocket makes contacts to the pheromone before it adopts a final conformation. However, we cannot discount that K260 may also, or instead, serve an alternative role such as stabilization of the CAP helix. In the case of the variable face, if that surface is mutated significantly reduced levels of transcription are observed (N220A Fig 8A) and the corresponding affinity of ComR for XIP is reduced by more than an order of magnitude (N220A Fig 9; Table 2). Moreover, wild-type *S*. *suis* ComR stimulated with *S*. *mutans* XIP did not activate *in vivo* transcription in the test-bed system (Fig 3 and S1 Fig) although we did observe binding *in vitro*. Interestingly, the *in vitro* interaction occurred with the same affinity and mechanism as the variable face mutant (N220A) titrated with the Suis XIP (Fig 9, Table 2). This shows that the conserved face provides contacts with XIP that are required for binding and the variable face provides contacts that provide further refinement of XIP selection. This is somewhat reminiscent of how the Rap proteins recognize their cognate peptides [47]. However, unlike the Rap family the ComR pheromone binding pocket positions the conserved and variable residues on opposite sides of the pheromone interface, and at least for the strict ComR proteins, a single residue substitution on the variable face does not appear sufficient to allow activation by heterologous XIPs (Fig 5, Fig 8 and Fig 9).

So how does ComR initially recognize and discriminate between possible peptide pheromones? A clue to this model comes directly from the crystal packing artifact observed in the crystal structure (Fig 6B). The N-terminus of ComR shares some features with its own XIP (specifically, tryptophan and glutamate side chains) and shows how a non-productive peptide might first fit into the apo-ComR pocket. We observe the W4 side-chain fitting into one of the likely aromatic-binding pockets with packing against K260. Although it makes some contacts with the variable face, it fails to make enough contacts with the conserved residues required for activity, especially in the back of the pocket (S5A and S5B Fig). We see that the residues N-terminal to M1 are swung outwards, making few contacts with ComR and fail to push into the back of the pocket to begin the conformational change for dimerization. One can hypothesize that the *S*. *mutans* peptide may fit into ComR *S*. *suis* in this manner, and without N220 to anchor the *S*. *suis* XIP it does not bind tight enough to lock in to the back of the pocket and induce activation. This model would allow for the initial binding of many different peptides, even those that are quite foreign like the promiscuous ComRs, yet allow for selectivity as demonstrated by the strict ComRs. Considering the biochemical data, pheromone recognition is ultimately accomplished by fine tuning the residues on the variable face. Furthermore, this artifact hints at how the unorthodox *S*. *suis* XIP might bind. The W4 position is likely the same as the N-terminal W of the C7 XIP, which would allow for the two C-terminal glutamate residues to fit into the electropositive back of the pocket to disrupt the interactions stabilizing the observed apo conformation and allow for the necessary conformational change.

In line with the structural and biochemical observations on XIP specificity, it is interesting to observe that in the work by Talagas et. al [37] the mechanism and affinity of type-I ComR interaction with pheromone differs in ITC experiments. For the type-III interaction studied here the affinity is ~800 nM and exothermic compared to ~40 nM for type-I and endothermic (Table 2). This contrast likely arises from the differences in the peptide sequence, namely that the *S*. *suis* XIP is charged and the *S*. *thermophilus* XIP is more hydrophobic. This suggests that the *S*. *suis* interaction is driven more by hydrogen bonding and electrostatics, whereas *S*. *thermophilus* is driven largely by the hydrophobic effect and entropy. We speculate that this most likely contributes to filtering of potential XIP peptides, but does not impact the overall mechanism of ComR activation. Additionally, given that the binding of XIP induces dimerization (S6 Fig, Talagas et. al. [37]), it was interesting to observe that a one-site model of the ITC data produced the best fit and expected ComR-XIP stoichiometry. This suggests that perhaps the binding of XIP to ComR is directly coupled and that the ComR-XIP monomer is an extremely short lived species

Taking the sum of our data into account, we could identify patterns associated with individual ComRs that displayed an intermediate selectivity. Using GBS 2603 as an example, we identified patterns within active XIPs to design agonists. As predicted, all three designed XIPs activated the GBS 2603 ComR, with two of the three peptides strongly inducing light activity. In this way, we were able to design ligand agonists without using receptor structural data. Whether these peptides function in the native strain to activate P_*comS*_ remains to be tested. Due to the finite number of active XIP variants tested of which we could compare to inactive variants, we were limited in determining specific residues that would improve an already productive interaction.

Here we have cataloged responses of several type-II ComR proteins to non-cognate XIPs and developed a model for how ComR selects, or filters for the proper XIP. Although we observed clear variations in response to heterologous pheromones among species, specificity did not appear to correlate with phylogenic relationship (diversity was apparent within species groups, e.g. *S*. *pyogenes*). The difficult question at hand is what does the XIP selectivity we observed mean for bacterial interactions occurring in nature? For example, could the promiscuous Bovis species activate its competence pathway upon detecting foreign XIP in its local environment? Likewise, is the control of competence in *S*. *mutans* so strictly regulated such that only Mutans-derived XIP will activate this trait? Both species are naturally found in complex microbial populations and the evolution of *comRS* to become either strict or promiscuous may provide an unseen advantage under some circumstances or in some communities. In particular, aside from the core competence regulon, SigX controls many other accessory genes [12] whose governance may impact fitness or interactions with other microorganisms. The limited size of our study made it difficult to correlate ComR specificity to virulence potential of individual strain isolates. For instance, while *S*. *pyogenes* strains MGAS5005 and MGAS315 are each documented as invasive strains and are of the most commonly isolated serotypes [48, 49], their observed responses to heterologous XIPs indicated distinct patterns, even though both alleles were categorized as intermediate. Larger sample sets are likely to be required to identify any associations between ComR specificity and phenotype, and with such analyses a more informative study, perhaps, would be to investigate correlations between ComR specificity and frequencies of horizontal gene transfer events, and might help to explain adaptability of strains or emergence of serotypes [50–52].

Although work remains to fully understand to what extent the ComRS system plays in species-cross communication in the regulation of natural competence, we have provided the ground work for studies aimed at revealing the role of ComRS in a myriad *Streptococcus* species, and have developed a detailed structural and biochemical model that describes how the quorum sensing receptor ComR recognizes and discriminates between pheromone molecules.

## Materials and Methods

### Bacterial strains, growth conditions and oligonucleotides

*S*. *mutans* strains were incubated at 37° C in anaerobic conditions (5% CO_2_) in Todd Hewitt Broth (THB; Difco) or in Chemically Defined Media (CDM; [53]) supplied with 1% (m/v) glucose and 20% glycerol for storage at -80° C. *E*. *coli* strains were incubated at 30° C or 37° C, as indicated, under aerobic conditions, in Luria Broth (LB; Difco). To select for strains, 0.75% agar was added to media with selective antibiotics and cultures were plated and incubated at either 30° C or 37° and under aerobic or anaerobic conditions, as indicated. Antibiotics used for selection of *S*. *mutans* strains were spectinomycin (200 μg mL^-1^) and erythromycin (1 μg mL^-1^). Antibiotics used for selection of *E*. *coli* strains were erythromycin (500 μg mL^-1^) and ampicillin (100 μg mL^-1^). Oligonucleotides were synthesized by IDT (Coralville, Iowa) and are listed in S1 Table.

### Construction of the ‘test-bed’ reporter strains

To construct *ΔcomRS*::*spec* ^*R*^ linear DNA, 1029 bps directly upstream of the *comR* (designated US-ComRS) encoding region as well as the 1097 bps directly downstream of the *comRS* inter-genic (IR) region (starting with the ATG site of *comS*; designated DS-ComR-IR) were amplified using the primer pairs LW4-26/LW6-46 and LW4-40/LW4-41, respectively. The LW6-46 primer contains a SalI tail while the LW4-40 contains a PstI tail. A 1009 bp product containing the spectinomycin resistance cassette (referred to as spec^R^) encoded within pLZ12 spec [54] was amplified using the primers LW2-33, containing a SalI cut site, and LW2-37, containing a PstI cut site. In a 1:1:1 molar ration, the spec^R^ segment was incubated with T4 DNA ligase (NEB) in-between the US-ComRS and the DS-ComR-IR segments at the SalI and PstI sites, respectively. To naturally transform *S*. *mutans* strain UA159 with the completed ligation reaction, we used the method described by Desai *et al*. [40] with modifications. Briefly, overnight cultures of *S*. *mutans* strain UA159 were diluted to an OD_600_ of 0.05 in 0.5 mL CDM + 1% (m/v) glucose and grown under anaerobic conditions at 37° C for 1 hour before adding 1 uM UA159 C7 XIP. Cultures were incubated an additional 1 hour at 37° C before addition of the ligation reaction. After 1 hour of incubation with DNA, cultures were plated on selective media and plates were incubated under anaerobic conditions at 37° C. Electro-competent cells from the resulting *S*. *mutans ΔcomRS*::*spec* strain (ES1) were made using a modified *S*. *pyogenes* preparation procedure [55]. Briefly, overnight cultures of ES1 were grown in THY + 1 mM glycine. The overnight cultures were diluted 1:20 in 50 mL THY + 1 mM glycine and grown until an OD_600_ of ~0.3 was reached. At that time, cells were immediately spun down to pellets before undergoing an additional spin after re-suspension in sterile deionized water. An additional wash step was done with 15% glycerol before storage of aliquots at -80°C. Electro-competent ES1 aliquots were electroporated with ~200 ng of purified ComR reporter plasmid and recovered in 1 mL THY at 37°C for 90 minutes. Cultures were plated on selective media and incubated at 37° under anaerobic conditions until colonies formed.

### Construction of reporter and expression plasmids

Reporter plasmid DNA was derived from genomic DNA isolated from *S*. *mutans* strain UA159, *S*. *pyogenes* strain MGAS5005, *S*. *pyogenes* strain MGAS315, *S*. *agalactiae* strain A909, *S*. *suis* clinical strain 01–18929, *S*. *agalactiae* strain 2603 v/r, *S*. *bovis* strain SboB, *S*. *bovis* strain Sbo 83, and *S*. *dysgalactiae* subsp. *equisimilis* strain ATCC35666. (S2 Table). Reporter plasmids were constructed either by restriction digest followed by ligation or by Gibson assembly as previously described [56] and as indicated in S3 Table. The plasmid insertions for pMRW101, pMRW103, pMRW104, pMRW105, pMRW107, pERB1, pERB2, pERB3, pERB4, pERB5, pERB6, and pERB7 were each amplified with primer pairs containing either a 5’-PstI tail and a 3’-SalI or -KpnI tail as indicated in S4 Table. The plasmid insertions for pERB20, pERB21, pERB22, pERB23, pERB24, pERB36, pERB37, pERB38, pERB39, pERB40, pERB41, and pERB42 were amplified using two segments of DNA amplified from pERB5 which overlapped the bp(s) mutated to alanine as listed in S4 Table. The *S*. *suis* mutant reporter plasmid backbone, pERB5, was linearized with PstI and KpnI and underwent Gibson Assembly with the two segments of DNA containing the overlap region harboring the bp mutation, as described by Gibson et al. [56]. *S*. *suis* wild type and mutant ComR expression plasmids (pERB15, pERB30, pERB31, pERB32, pERB33, pERB34, pERB43, pERB44, pERB45, pERB46, pERB47, pERB48, pERB51) were synthesized using Gibson assembly after amplification of the wild type or mutated allele from the respective reporter plasmid (S4 Table) using primers EB147/EB148 containing 5’ and 3’ overlapping regions allowing recombination with the expression vector pET-15b (Novagen, Billerica, MA). Electro-competent cells were prepared from *E*. *coli* strain BH10C [57] and BL21(DE3) [58]. De-salted ligation reactions and 1:3 dilutions of Gibson assembly reactions were transferred into electro-competent cells. After electroporation, cells were recovered in LB, at 30° C for 90 minutes before plating and incubation at 30° C. All constructs were confirmed by DNA sequencing.

### Preparation of synthetic peptides

Predicted XIP peptide sequences were determined by identifying open reading frames downstream from the putative ComR gene by using NCBI BLAST to identify UA159 ComR homologues. One to three different lengths of peptide from each species were ordered from NeoScientific (Woburn, MA). Peptide crude extracts (purity 45–75%) were dissolved in DMSO (Fisher Scientific, Hampton, NH) at a concentration of 1 mM based upon the specific purity and stored at -20° C.

### Test-bed reporter assays

Starter cultures, from cultures stored in 20% glycerol at -80° C, of each ComR reporter strain were diluted to an OD_600_ of 0.05 in CDM + erm and incubated at 37° C until an OD^600^ of ~0.1. 190 uL aliquots of reporter strain cell culture were added to wells within a 96-well (Greiner Bio-One; Monroe, NC) lidded, clear, flat-bottom plate, each containing 10 μL of DMSO or a 5x-serial dilution of the peptide of interest in DMSO (final concentration of 5% DMSO/well). Concentrations tested for each peptide were 1.3 nM, 6.4 nM, 38.6 nM, 231.5 nM, 1388.9 nM, 83.3 μM, and 50 μM. 50 μL of a 1% decanal solution in mineral oil was added between each well in the plate to provide substrate for the luciferase reporter. Prepared plates were lidded and sealed with Parafilm (Bemis, Oshkosh, WI) before incubation at 37° C, with continuous shaking, in a Synergy 2 plate reader (Biotek). OD_600_ and luminescence readings were measured every 15 min. over a 4-hr period. The maximum RLU measurement per well, between 60–240 minutes was recorded using Gen5 data analysis software. For each well, baseline-corrected induction curves, EC50 values, and significance values between 50 μM and 0 μM peptide treatment (p <0.05) were calculated to determine the activity profile of each ComR (GraphPad, La Jolla, CA).

### Protein Expression and Purification

ComR *S*. *suis* 05ZYH33 (residues 1–304) was cloned as an N-terminal 6-his tag in the vector pET15b. BL21(DE3) cells were transformed with the expression vector and grown in LB media at 37°C until an OD of > 0.6 at 600 nm with protein expression induced by the addition of 1 mM isopropyl β-D-1-thiogalactopyranoside (IPTG). The temperature was reduced to 20°C and cultures were allowed to grow overnight. Cells were harvested by centrifugation followed by lysis with an Emulsiflex-C5 (Avestin). The lysate was cleared by centrifugation at 16,000 rpm for 30 minutes and then passed over a nickel NTA gravity column (Pierce) followed by a wash with 50 column volumes of chilled buffer (50 mM Tris pH 7.5, 500 mM NaCl, 25 mM imidazole). The protein was eluted with 5 column volumes elution buffer (50 mM Tris pH 7.5, 500 mM NaCl, 500 mM imidazole) and further purified using an SD75 16/60 superdex gel filtration column (GE Healthcare) via AKTA (GE Healthcare) at 4°C in a final buffer of 20 mM Tris pH 7.5, 100 mM NaCl, 1 mM beta-mercaptoethanol (β-ME). Selenomethionine labeled protein was produced using metabolic inhibition [59]. Briefly, cells were grown in M9 media at 37°C followed by reducing the temperature to 20°C and adding 0.05 g/L seleomethionine, leucine, valine, proline, and 0.1 g/L lysine, threonine, phenylalanine. The cells were allowed to grow for an additional 30 minutes before induction with 1 mM IPTG. All ComR protein variants were created by quick-change mutagenesis (Strategene) and expressed and purified similar to wild-type.

### Protein Crystallization

ComR *S*. *suis* 05ZYH33 was concentrated to 25 mg/mL for initial screening in commercially available conditions with a Tecan Freedom Evo 200 robot at the University of Illinois at Chicago Research Resources Center High Through-put facility. The crystallization conditions were 10 to 25 mg/mL ComR with a 1:1 mixture of 15% PEG 3350, 0.2 M sodium citrate. Crystals were grown by sitting drop vapor diffusion at 4°C with micro-seeding using SeedBead (Hampton Research).

### Data collection and refinement

Diffraction data was collected at the Advanced Photon Source at Argonne National Laboratories as part of the LS-CAT, Sector 21. Protein crystals were prepared by overnight dehydration in mother liquor substituted with 15% PEG8000 against 0.2 M sodium citrate and 35% PEG8000 followed by flash freezing in liquid nitrogen. Data was processed using XDS [60] and phases determined by single-wavelength anomalous dispersion (SAD) on data collected near the selenium peak using both the Phenix package [61] and Sharp/Autosharp [62, 63]. The initial model was further built and refined using Coot [64], Refmac5 [65] from the CCP4 suite of programs [66], Phenix [61], and TLS refinement [67]. PDB_REDO was used for final validation and refinement [68]. The final model has an R/Rfree of 22.0/25.5% with 99.7% of residues in the allowed region of the Ramachandran plot. The coordinates and structure factors (code 5FD4) have been deposited to the Protein Data Bank, Research Collaboratory for Structural bioinformatics, Rutgers University, New Brunswick, NJ (www.rcsb.org and www.pdb.org).

### Circular Dichroism Spectroscopy

Purified ComR *S*. *suis* and variant proteins were diluted into 20 mM KHPO_4_ at pH 7.5 in a 0.2 cm path-length cuvette. Spectra were collected on a Jasco-815 spectropolarimeter from a wavelength of 260 nm to 190 nm. Background spectra of the buffer diluted into 20 mM KHPO_4_ was subtracted from the data. To plot the data as mean residue ellipticity, a mean residue weight of 118.1 was used with protein concentrations of 0.05 mg/mL for wild-type, 0.01 mg/mL for K260A, 0.03 mg/mL for N220A, R103A, and Q40A.

### Isothermal Titration Calorimetry

Purified ComR *S*. *suis* and variant proteins (K260A, N220A, and Q40A) were dialyzed overnight at 4°C into the same buffer stock of 20 mM Tris pH 7.5 100 mM NaCl 1 mM β-ME. HPLC purified synthetic peptides for both *S*. *suis* 05ZYH33 (WGTWVEE) and *S*. *mutans* U159 (GLDWWSL) were obtained from NeoScientific (Woburn, MA). Each XIP was reconstituted in the experimental buffer followed by centrifugation at 12,000 rpm to clear undissolved or precipitating material. XIP was used at 300 μM concentration and injected into 20 μM ComR. All experiments were performed using a VP-ITC calorimeter (Malvern) at 25°C. Controls included titration of XIP into buffer alone or the titration of buffer into ComR. The final heats of binding were analyzed using Origin Software (Malvern) using a one-site model.

### Dynamic Light Scattering

Purified ComR *S*. *suis* and protein variants were injected into a DynaPro-801 (Protein Solutions) using a syringe with a 0.1 μm filter in 20 mM Tris pH 7.5 100 mM NaCl 1 mM β-ME after centrifugation at 6000 rpm for 15 minutes to remove any larger precipitates. The concentration of ComR was ~ 20 μM except for Q40A which was diluted to ~ 5 μM due to increased signal from aggregates. XIP was added to wild-type ComR at a concentration of 50 μM. Data was analyzed with the provided software as mono-modal or bi-modal using an aqueous buffer model.

## Supporting Information

S1 Fig Individual ComR activity with each cognate and non-cognate XIP variant when expressed the *S*. *mutans* ES1 background. Bar graphs (top) indicate the percent maximal induction of ComR when treated with 50 μM of the indicated XIP variant when normalized to the cognate XIP eliciting the greatest increase (arrow). XY graphs (bottom) display titration curves of each peptide eliciting a response with ComR as observed from 50 uM peptide treatment (from bar graph). *Peptides were not titrated to sufficiently low concentrations to determine EC50 values.
(A)
*S*. *agalactiae* 2603 v/r ComR.(B)
*S*. *dysgalactiae* subsp. *equisimilis* ATCC35666 ComR.(C)
*S*. *pyogenes* MGAS315 ComR.(D)
*S*. *pyogenes* MGAS5005 ComR.(E)
*S*. *bovis* 83 ComR.(F)
*S*. *bovis* SboB ComR.(G)
*S*. *suis* 05ZYH33 ComR.(H)
*S*. *mutans* UA159 ComR.(I)
*S*. *agalactiae* A909 ComR.
(TIF)

S2 Fig Apo-ComR *S*. *suis* is a monomer in solution. Purified ComR *S*. *suis* was assayed by size-exclusion chromatography at high-concentrations (~1 mM) using an SD75 column (GE healthcare) equilibrated in 20 mM Tris pH 7.5 100 mM NaCl 1 mM β-ME. The monomer elutes at approximately 60 mL. The inlay shows the molecular weight standards and the calculated weight of ComR *S*. *suis*. The predicted stokes radius is 2.84 nm with these standards.
(TIF)

S3 Fig **Crystal packing and data quality of the native and derivative data set** (A) The asymmetric unit of native ComR is shown as a cartoon with monomer A colored red and monomer B colored tan. (B) The asymmetric unit of selenomethionine substituted ComR is shown as a cartoon in red and a symmetry mate in tan. (C) Structural alignment of the native structure and unrefined selenomethionine substituted structure, rmsd = 0.69 Å^2^. (D) The electron density for helices 15 and 16 is shown for the native (left) and derivative (right) using Coot, contoured at 1.1 rmsd (0.126 e/Å^3^). The DBD and TPR and labeled in addition to helices 15 and 16.
(TIF)

S4 Fig Structural comparison of the XIP binding pocket between strict and promiscuous recognition ComR proteins. The program Modeller was used to create homology models of all ComR proteins in this study using the apo *S*. *suis* structure as a template. Two-strict and two promiscuous models are presented here to show general trends (A) Electrostatic surface potential calculated by APBS (-10 kTe to 10 kT/e), blue indicating electropositive and red indicating electronegative. (B) hydrophobic character of the pocket is plotted, with hydrophobic residues in orange and aromatic residues in purple. (C) Shape, volume and surface area of the XIP pocket as determined by V3 server (http://3vee.molmovdb.org/.) Two views of each pocket are shown as a 180 rotation. Left is looking into the pocket and right looking out of the pocket. The total volume and surface area is listed. The aliphatic index of the variable face is also listed.
(TIF)

S5 Fig Species comparison of the ComR XIP binding pocket. (A) View of the XIP binding pocket of *S*. *suis* compared to *S*. *thermophilus*. *S*. *suis* is shown in red with regions of differing conformation shown in beige (*S*. *thermophilus* apo) and blue (*S*. *thermophilus* activated). ComS is shown in green and residues that contact XIP or the artifact peptide indicated by color (*S*. *suis* in red, *S*. *thermophilus*, blue). (B) Comparison of the artifact peptide and ComS. A molecular surface representation of ComR *S*. *suis* is shown with the artifact peptide in yellow and ComS *S*. *thermophilus* in green. Residues of ComR *S*. *suis* selected for mutation are indicated. (C) Alignment of the TPR of *S*. *suis*, *S*. *thermophilus* and modeled type-II ComR proteins used in this study. All Asn residues for the selected species in the TPR are shown and indicated by both color and position (*S*. *suis* numbering).
(TIF)

S6 Fig Dynamic Light Scattering of ComR variants and XIP. (A) Wild-type apo ComR *S*. *suis* is a monomer. (B) In the presence of a 2.5-fold excess of cognate XIP, ComR particle size increases to form a dimer. (C) The variable face variant N220A is a monomer in solution. (D) The DBD-TPR interface variant self-associates in solution to form a dimer and larger aggregates. Wild-type and N220A were measured at 20 μM protein concentration and 50 μM XIP added in panel B to observe the wild-type dimer. Q40A was measured at 5 μM due to increased signal from larger aggregates. The buffer used was 20 mM Tris pH 7.5 100 mM NaCl 1 mM β-ME. The radius reported in the figure is from the fit of the histogram as a weighted average of all observed species. Both the wild-type and the N220A variant show a species (~40–60% total mass) with a radius of approximately 2.6 nm in agreement with SEC (S2 Fig). The major species for wild-type + XIP is 3.9 nm (47% total mass) and the Q40A variant are approximately 3.9 nm (24% total mass) and 5.3 nm (55% total mass).
(TIF)

S1 Table Primers used in this study. (XLSX)

S2 Table *S*. *mutans* UA159 derived strains used in study. (XLSX)

S3 Table Plsmids used in this study. (XLSX)

S4 Table Plasmid construction strategies used in this study. (XLSX)
