# Supplementary figures and images for: Pheromone Recognition and Selectivity by ComR Proteins among Streptococcus Species
Source: PLoS Pathog. 2016 Dec 1;12(12):e1005979. doi: 10.1371/journal.ppat.1005979 (PMC5131902; doi:10.1371/journal.ppat.1005979)

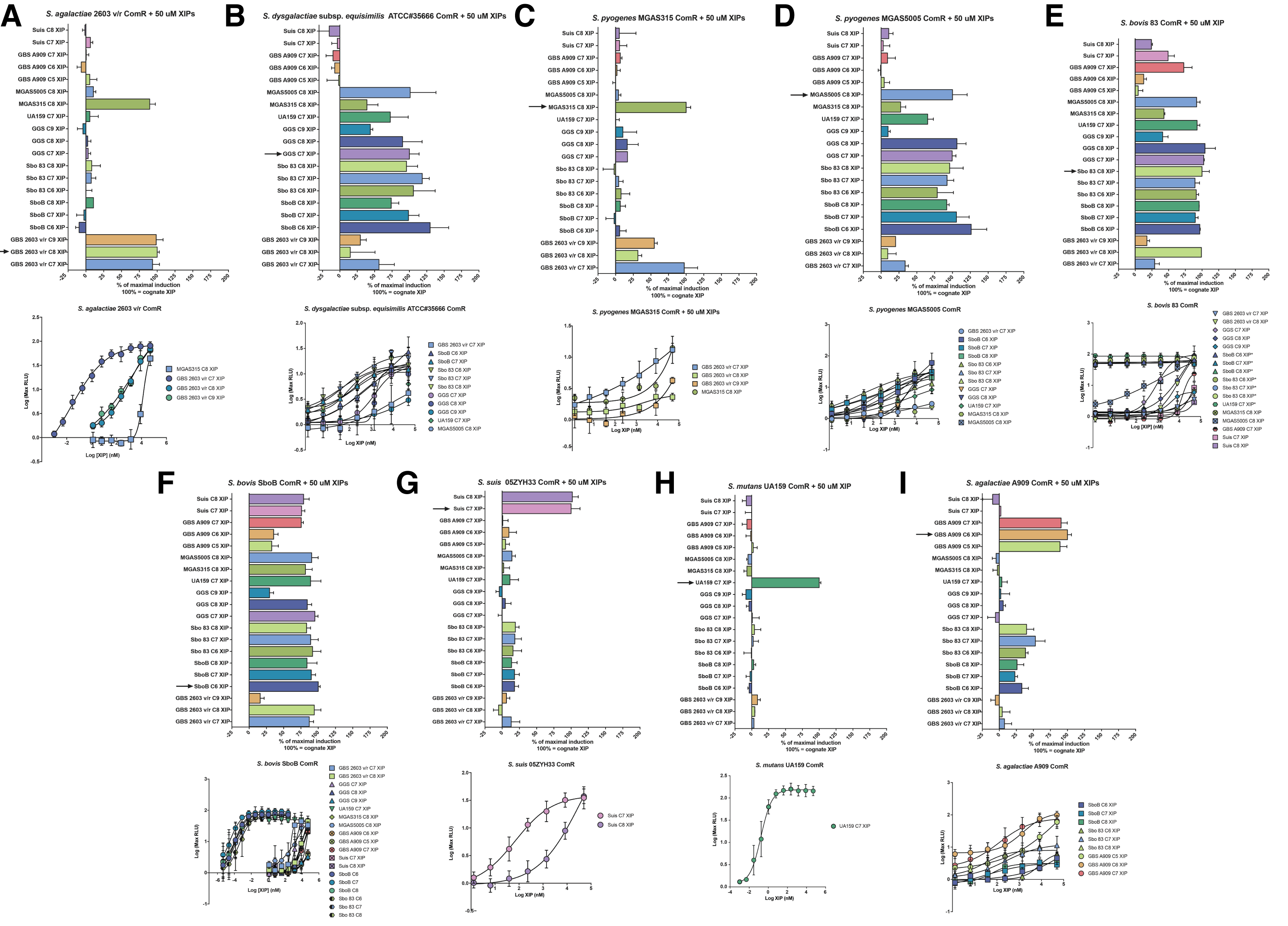

Supplement: S1 Fig — Bar graphs (top) indicate the percent maximal induction of ComR when treated with 50 μM of the indicated XIP variant when normalized to the cognate XIP eliciting the greatest increase (arrow). XY graphs (bottom) display titration curves of each peptide eliciting a response with ComR as observed from 50 uM peptide treatment (from bar graph). *Peptides were not titrated to sufficiently low concentrations to determine EC50 values. (A) S. agalactiae 2603 v/r ComR.(B) S. dysgalactiae subsp. equisimilis ATCC35666 ComR.(C) S. pyogenes MGAS315 ComR.(D) S. pyogenes MGAS5005 ComR.(E) S. bovis 83 ComR.(F) S. bovis SboB ComR.(G) S. suis 05ZYH33 ComR.(H) S. mutans UA159 ComR.(I) S. agalactiae A909 ComR. (TIF) [file ppat.1005979.s001.tif]

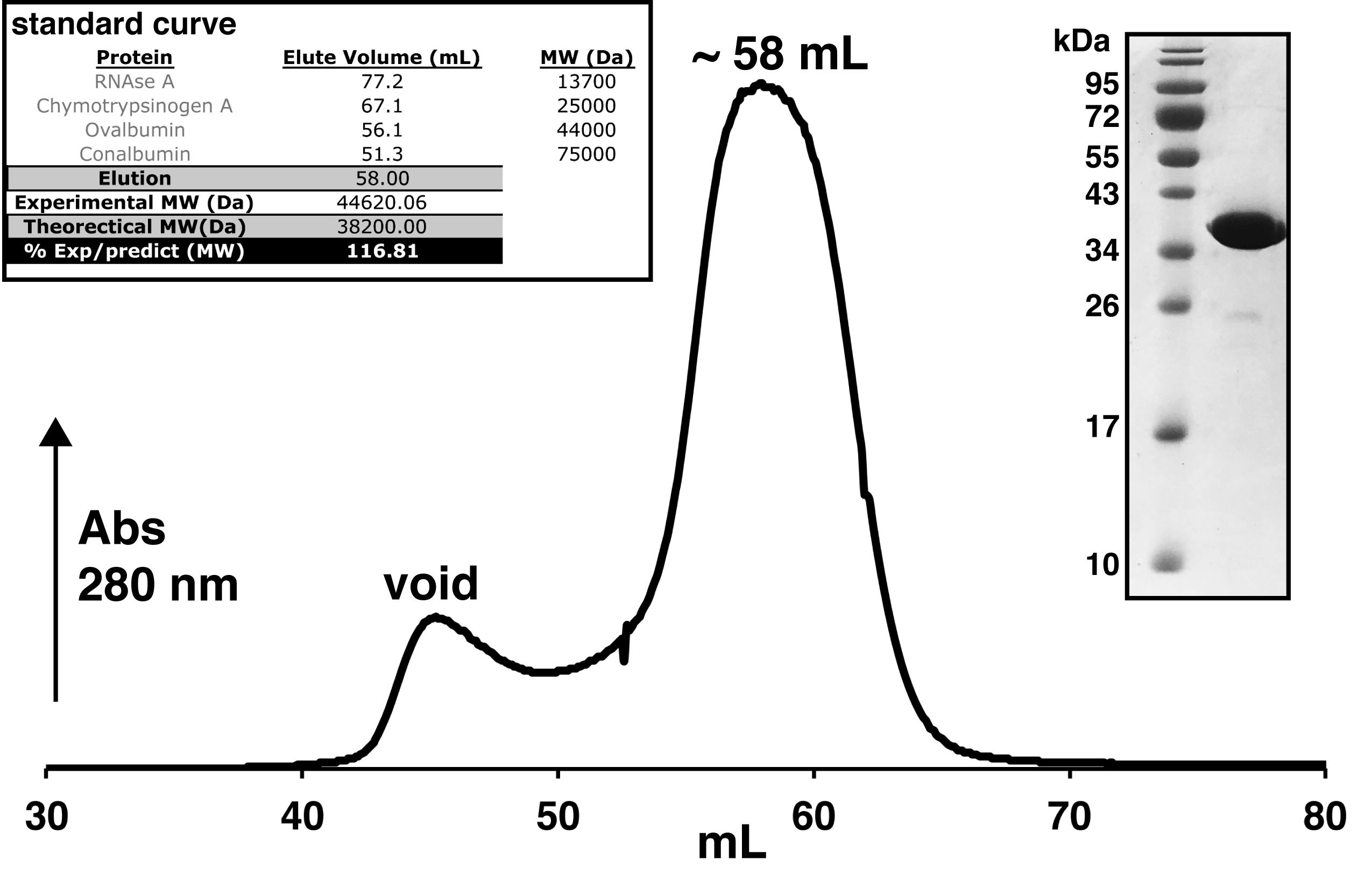

Supplement: S2 Fig — Purified ComR S. suis was assayed by size-exclusion chromatography at high-concentrations (~1 mM) using an SD75 column (GE healthcare) equilibrated in 20 mM Tris pH 7.5 100 mM NaCl 1 mM β-ME. The monomer elutes at approximately 60 mL. The inlay shows the molecular weight standards and the calculated weight of ComR S. suis. The predicted stokes radius is 2.84 nm with these standards. (TIF) [file ppat.1005979.s002.tif]

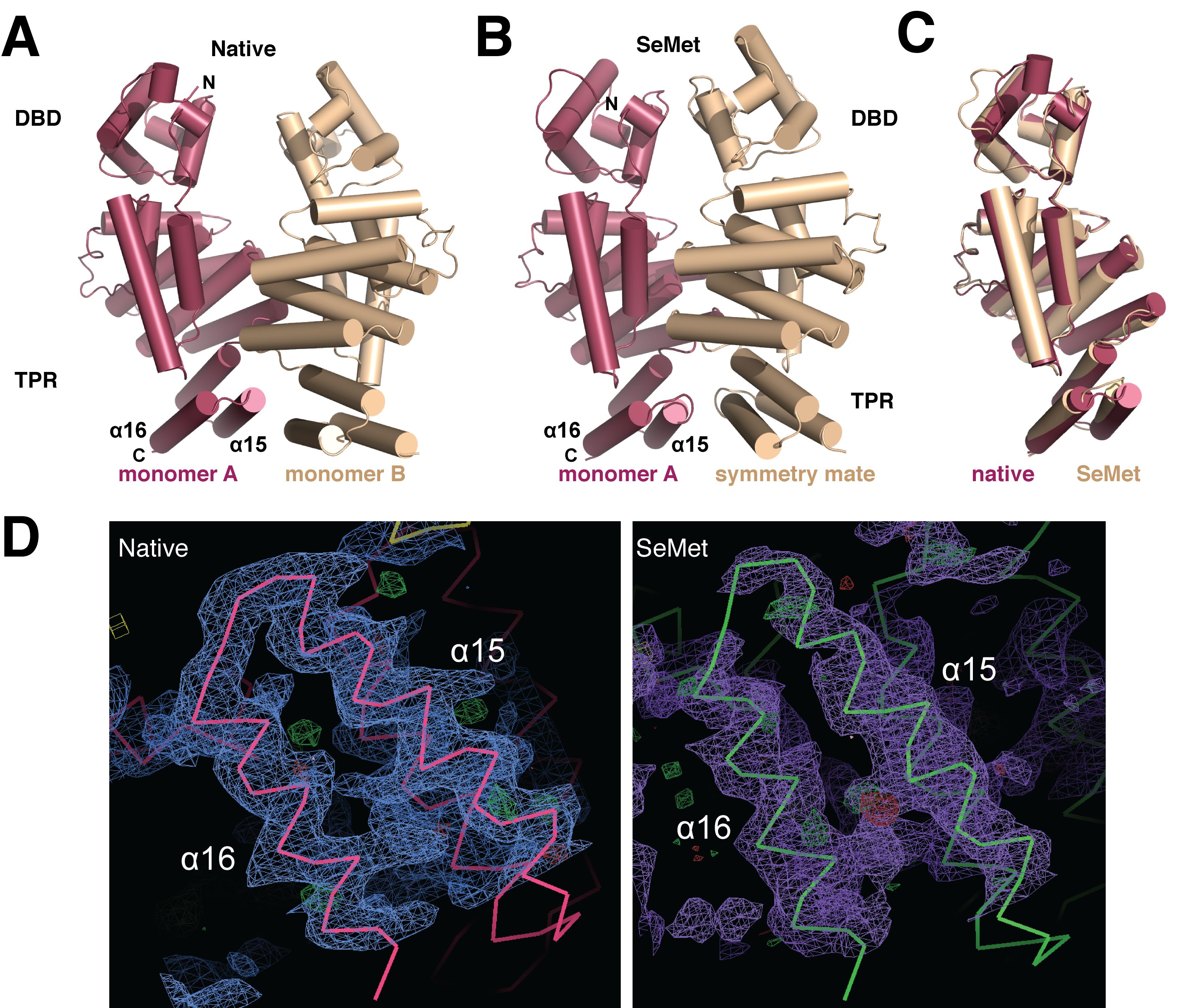

Supplement: S3 Fig — Crystal packing and data quality of the native and derivative data set (A) The asymmetric unit of native ComR is shown as a cartoon with monomer A colored red and monomer B colored tan. (B) The asymmetric unit of selenomethionine substituted ComR is shown as a cartoon in red and a symmetry mate in tan. (C) Structural alignment of the native structure and unrefined selenomethionine substituted structure, rmsd = 0.69 Å2. (D) The electron density for helices 15 and 16 is shown for the native (left) and derivative (right) using Coot, contoured at 1.1 rmsd (0.126 e/Å3). The DBD and TPR and labeled in addition to helices 15 and 16. (TIF) [file ppat.1005979.s003.tif]

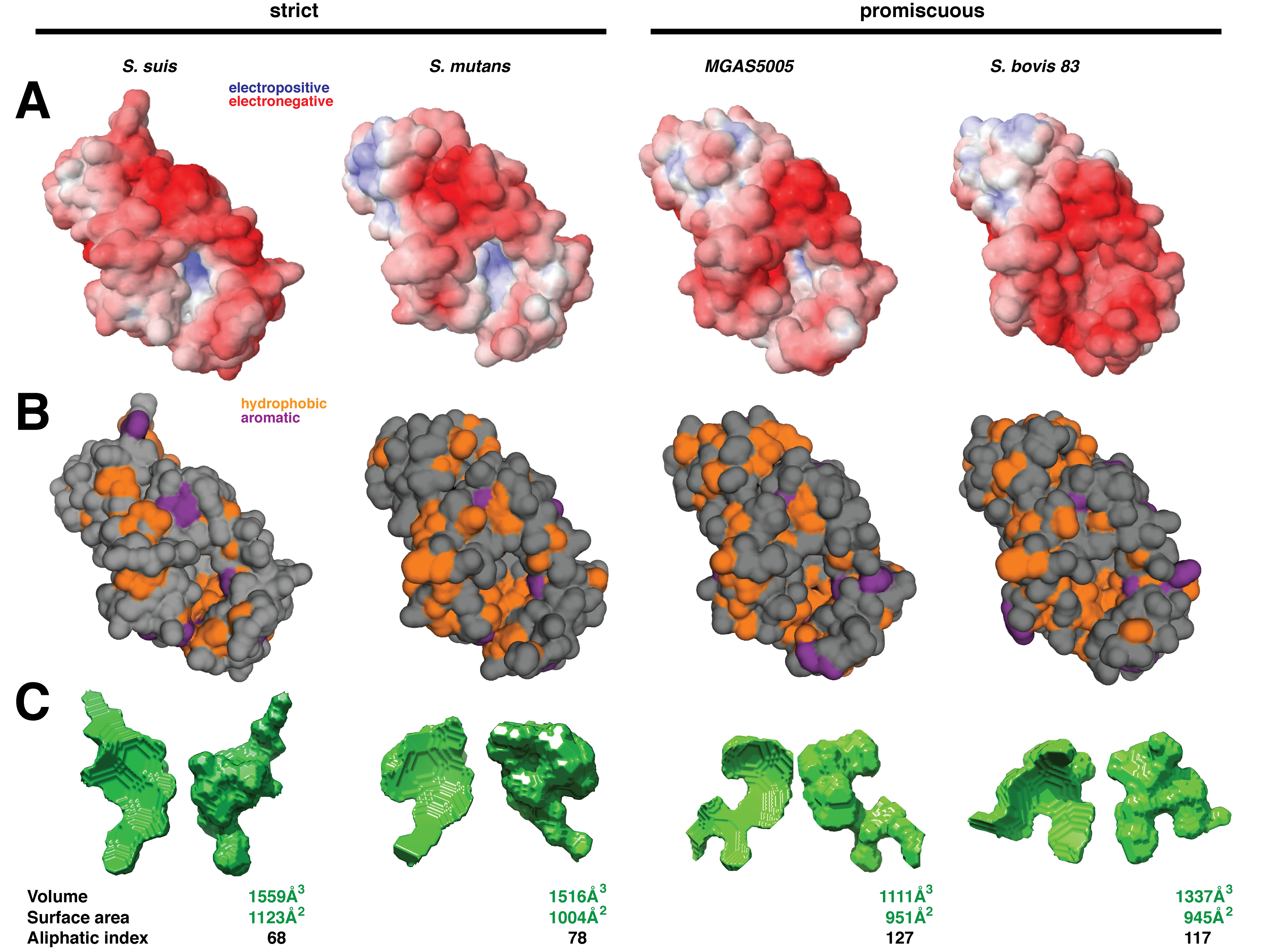

Supplement: S4 Fig — The program Modeller was used to create homology models of all ComR proteins in this study using the apo S. suis structure as a template. Two-strict and two promiscuous models are presented here to show general trends (A) Electrostatic surface potential calculated by APBS (-10 kTe to 10 kT/e), blue indicating electropositive and red indicating electronegative. (B) hydrophobic character of the pocket is plotted, with hydrophobic residues in orange and aromatic residues in purple. (C) Shape, volume and surface area of the XIP pocket as determined by V3 server (http://3vee.molmovdb.org/.) Two views of each pocket are shown as a 180 rotation. Left is looking into the pocket and right looking out of the pocket. The total volume and surface area is listed. The aliphatic index of the variable face is also listed. (TIF) [file ppat.1005979.s004.tif]

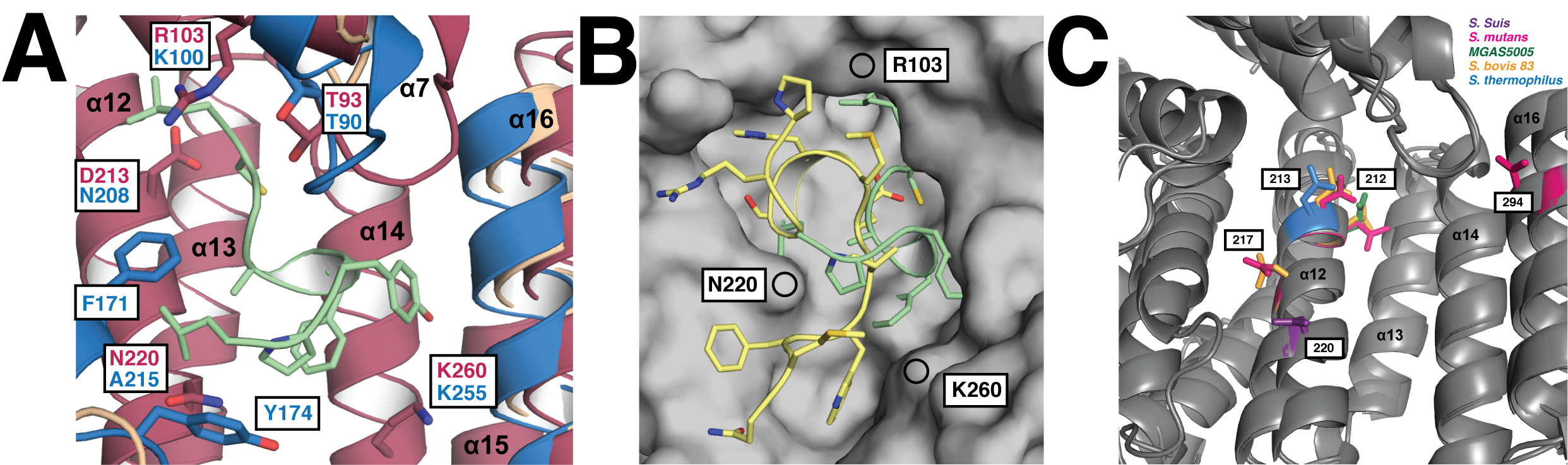

Supplement: S5 Fig — (A) View of the XIP binding pocket of S. suis compared to S. thermophilus. S. suis is shown in red with regions of differing conformation shown in beige (S. thermophilus apo) and blue (S. thermophilus activated). ComS is shown in green and residues that contact XIP or the artifact peptide indicated by color (S. suis in red, S. thermophilus, blue). (B) Comparison of the artifact peptide and ComS. A molecular surface representation of ComR S. suis is shown with the artifact peptide in yellow and ComS S. thermophilus in green. Residues of ComR S. suis selected for mutation are indicated. (C) Alignment of the TPR of S. suis, S. thermophilus and modeled type-II ComR proteins used in this study. All Asn residues for the selected species in the TPR are shown and indicated by both color and position (S. suis numbering). (TIF) [file ppat.1005979.s005.tif]

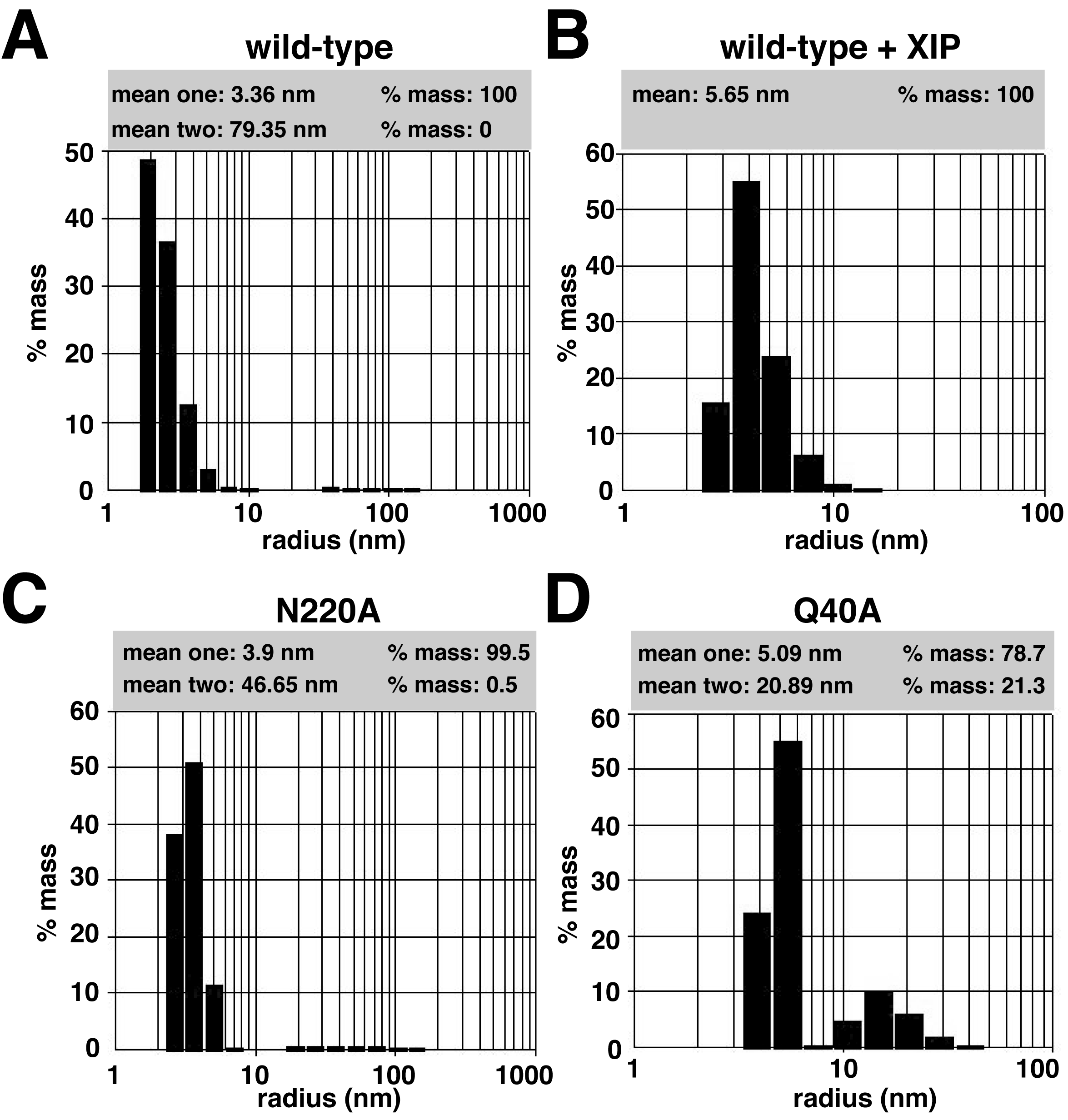

Supplement: S6 Fig — (A) Wild-type apo ComR S. suis is a monomer. (B) In the presence of a 2.5-fold excess of cognate XIP, ComR particle size increases to form a dimer. (C) The variable face variant N220A is a monomer in solution. (D) The DBD-TPR interface variant self-associates in solution to form a dimer and larger aggregates. Wild-type and N220A were measured at 20 μM protein concentration and 50 μM XIP added in panel B to observe the wild-type dimer. Q40A was measured at 5 μM due to increased signal from larger aggregates. The buffer used was 20 mM Tris pH 7.5 100 mM NaCl 1 mM β-ME. The radius reported in the figure is from the fit of the histogram as a weighted average of all observed species. Both the wild-type and the N220A variant show a species (~40–60% total mass) with a radius of approximately 2.6 nm in agreement with SEC (S2 Fig). The major species for wild-type + XIP is 3.9 nm (47% total mass) and the Q40A variant are approximately 3.9 nm (24% total mass) and 5.3 nm (55% total mass). (TIF) [file ppat.1005979.s006.tif]
